# The strong propensity of Cadherin‐23 for aggregation inhibits cell migration

**DOI:** 10.1002/1878-0261.12469

**Published:** 2019-03-19

**Authors:** Malay K. Sannigrahi, Cheerneni S. Srinivas, Nilesh Deokate, Sabyasachi Rakshit

**Affiliations:** ^1^ Department of Chemical Sciences Indian Institute of Science Education and Research Mohali Chandigarh India; ^2^ Department of Biological Sciences Indian Institute of Science Education and Research Mohali Chandigarh India; ^3^ Centre for Protein Science Design and Engineering Indian Institute of Science Education and Research Mohali Chandigarh India

**Keywords:** cadherin‐23, cell migration, cell–cell adhesion, lung cancer, non‐classical cadherins, promoter methylation, soluble isoforms

## Abstract

Cadherin‐23 (Cdh23), a long‐chain non‐classical cadherin, exhibits strong homophilic and heterophilic binding. The physiological relevance of strong heterophilic binding with protocadherin‐15 at neuroepithelial tip links is well‐studied. However, the role of Cdh23 homodimers in physiology is less understood, despite its widespread expression at the cell boundaries of various human and mouse tissues, including kidney, muscle, testes, and heart. Here, we performed immunofluorescence studies that revealed that Cdh23 is present as distinct puncta at the cell–cell boundaries of cancer cells. Analysis of patient data and quantitative estimation of Cdh23 in human tissues (normal and tumor) also indicated that Cdh23 is down‐regulated via promoter methylation in lung adenocarcinoma (AD) and esophageal squamous cell carcinoma (SCC) cells; we also observed a clear inverse correlation between Cdh23 expression and cancer metastasis. Using HEK293T cells and four types of cancer cells differentially expressing Cdh23, we observed that cell migration was faster in cells with reduced levels of Cdh23 expression. The cell migration rate in cancer cells is further accelerated by the presence of excretory isoforms of Cdh23, which loosen its cell‐adhesion ability by competitive binding. Overall, our data indicate the role of Cdh23 as a suppressor of cell migration.

**Abbreviations**
AJ: adherin junction
CAM: cell adhesion molecules
Cdh23: cadherin‐23
CI: confidence interval
Ecdh: E‐cadherin
EC: extracellular
ESCC: esophageal squamous cell carcinoma
ESTA: esophageal squamous cell carcinoma and metastasis array
HR: hazard ratio
IF: immunofluorescence
IHC: immunohistochemistry
LC: lung cancer
LCTA: lung cancer progression tissue array
LDH: lactate dehydrogenase
LUAD: lung adenocarcinoma
MRE: mean relative expression
pCdh23: plasmid cadherin‐23
qRT‐PCR: quantitative real‐time PCR
RSD: relative standard deviation
RSEM: RNA‐Seq by expectation maximization
SD: standard deviation
SEM: standard error of the mean
SMA: subapical membrane apposition
TCGA: The Cancer Genome Atlas
THPA: The Human Protein Atlas
TMA: tissue microarray

## 1 Introduction

Cadherins mediate Ca^2+^‐dependent cell–cell adhesion and regulate embryogenesis (Tepass *et al*., 2000), tissue architecture (Halbleib and Nelson, 2006), cell proliferation (Jeanes *et al*., 2008), and signal transductions (Klezovitch and Vasioukhin, 2015) in multicellular organizations. Since the extracellular (EC) region along with transmembrane and intracellular parts, comprise cadherins, they respond to external cues and regulate various intracellular pathways (Halbleib and Nelson, 2006; Klezovitch and Vasioukhin, 2015). However, many of these phenomena are predominantly described by classical cadherins (Niessen *et al*., 2011) forming adherin junctions (AJ) or desmosomal cadherins in desmosomal junctions (Tanoue and Takeichi, 2004), partly due to their high level of endogenous expression in diverse tissues. The EC region of these cadherins is extended to only five domains, and forms the intercellular junctions 20–25 nm wide at the less flexible membranous region (Chen and Wu, 2017; Tsukasaki *et al*., 2014). There are other types of cadherins, members of the non‐classical cadherin family, with long EC regions, e.g. mammalian Fat cadherins with 34 EC domains, mammalian Dachsous cadherins and cadherin‐23 (Cdh23) with 27 EC domains, and more. Endogenous expression of these long cadherins is relatively low in higher vertebrates, and their role in physiology is mostly unexplored. Until recently, an exploration of the high‐resolution structure of cell–cell boundaries has distinctly identified an additional cell–cell junction, ‘subapical membrane apposition’ (SMA), along with AJs. The SMA is constructed by the heterophilic interactions between Fat cadherins and Dachsous cadherins with extended overlap between multiple EC domains. The long overlap interface in the heterophilic interaction mediates stronger binding and thus facilitates the adhesion between flexible membranes with an intercellular distance of 40–50 nm (Chen and Wu, 2017; Tsukasaki *et al*., 2014). There is a similar heterophilic interaction between Cdh23 and protocadherin‐15 (another long‐chain non‐classical cadherin) at the tips of two adjacent stereocilia in neuroepithelial cells, known as tip‐links. Tip‐links as gating‐springs, too, withstand strong mechanical tension from sound in hearing (Kazmierczak *et al*., 2007).

Apart from heterophilic interactions, Cdh23 is known to participate in homophilic interactions and has also demonstrated stronger cell aggregation ability *in vitro* (Cohen *et al*., 2017; Siemens *et al*., 2004). Non‐aggregating L929 cells expressing either recombinant full‐length Cdh23 or E‐cadherin (Ecdh) showed explicit sorting of cells with distinct Cdh23‐ and Ecdh‐mediated cell aggregates, indicating their unique and explicit nature comprising homophilic interactions (Siemens *et al*., 2004). However, the physiological significance of the homophilic binding of Cdh23 is yet to be explored. The Human Protein Atlas (THPA) has reported the expression of Cdh23 in 97.7% of tissues (43 of 44 tissues except for liver; Fig. S1a,b). *In vitro* studies with the MCF‐7 breast cancer cell‐line showed the localization of Cdh23 at the cell–cell junction, similar to classical cadherins (Apostolopoulou and Ligon, 2012). In addition, Cdh23 was identified at the contacts between MCF7 and normal breast fibroblasts (NBF), fibroblasts leading to heterotypic cell–cell junctions. In both these junctions, the cell–cell contacts are mediated by the homophilic interactions of Cdh23 from opposing cell surfaces (Apostolopoulou and Ligon, 2012). The genome‐wide association studies of kidney function identified a strong association of Cdh23 with the estimated cross‐sectional glomerular filtration rate, and its knockdown in zebrafish embryo resulted in severe edema, suggesting its essential role in normal kidney functions (Gorski *et al*., 2015). Not only is Cdh23 differentially expressed in various tissues, but these proteins are also tightly regulated in diseases. The Cancer Genome Atlas (TCGA) marks the significant down‐regulation of Cdh23‐mRNA in 24 types (65%) of cancers among 37 types and up‐regulation in four types (Fig. S1c). The extent of down‐regulation is greater than in other cadherins and was mostly observed in solid cancers (Fig. S1c,d).

We hypothesized that the down‐regulation of Cdh23 during tumorigenesis weakens the cell–cell junction and promotes cancer cell migration leading to metastasis. Based on this hypothesis, here we first established the widespread expression of Cdh23 in different normal and cancer tissues using *in silico* and experimental approaches, and deciphered its down‐regulation in cancer. Next, we explored how downregulation of Cdh23 affects the cellular functionalities and contributes towards metastasis. We also identified a strong correlation of Cdh23‐expression with metastasis and patient survival.

## 2 Material and methods

### 2.1 Patient specimens and cell cultures

Tissue microarrays (TMA) of cancer biopsy samples were procured: lung cancer progression tissue array (LCTA; LC1005a; Biomax, Rockville, MD, USA) and esophagus squamous cell carcinoma and metastatic carcinoma tissue array (ES2001; Biomax). They were stained using Dako REAL EnVision Detection System kit (K500711; Dako, Glostrup, Denmark).

Ten different cancer cell‐lines were procured from NCCS (Pune, India): namely, HeLa, HaCat, HEK293T, A549, KB, Hep‐2, MCF‐7, L132, PC‐3, and WRL68.

### 2.2 RNA isolation and real‐time quantitative PCR

Total RNA was isolated from cell lines using RNA isolation kit (Bio‐Rad, München, Germany). Total RNA (1 μg) was treated with DNase using DNase I Amplification Grade kit (AMPD1; Sigma, ?St. Louis, MO, USA) and used for cDNA synthesis using mRNA first‐strand cDNA synthesis kit (Bio‐Rad). The resulting cDNA products were stored at −20 °C. qPCR was performed using Cdh23 specific primers (Tables S1 and S2) in the CFX96 Real‐Time PCR Detection System (Bio‐Rad).

### 2.3 Western blot, immunohistochemical staining (IHC), immunofluorescence (IF)

Western blotting was performed on the cell‐lines using standard protocols and IHC on TMA using Dako REAL EnVision Detection System kit (K500711; Dako) (Coventry *et al*., 1995; Hirano, 2012). Antibodies used for IHC are Cdh23 (HPA017232; Sigma) and Ecdh (HPA004812; Sigma). Moreover, Cdh23 N‐terminal (PA5‐43398; Thermo Fisher Scientific, ?Waltham, MA?, USA), β‐catenin (sc‐7963; Santa Cruz, Santa Cruz, CA, USA) and anti‐β‐actin (A1978; Sigma) specific antibodies were used for western blotting. For IF studies, anti‐mouse IGG (H + L), CF594 (SAB4600092; Sigma) and anti‐rabbit IGG (H + L), CF633 (SAB4600141; Sigma) were used as secondary antibodies. Wnt/β‐Catenin downstream activated targets were identified using Antibody Sampler Kit #8655 (Cell Signaling Technology, Danvers, MA, USA).

### 2.4 Transfection with cadherin 23 plasmid (pCdh23) and siRNA/scrambled control

Cadherin‐23 plasmid (pCDH23) was a gift from R. Ladher (NCBS, Bangalore, India).

Cdh23‐siRNA (AM16708; Thermo Fisher Scientific) was used to inhibit endogenous Cdh23 expression. Transfection was done using Lipofectamine 2000 (Catalog #11668; Thermo Fisher Scientific, Carlsbad, CA, USA). Cells were treated with 50, 100, and 200 pmol·mL^−1^ of siRNA. Scrambled siRNA (Catalog # 4464058; Thermo Fisher Scientific) was used as negative control. pLKO.1 puro shRNA Ecdh was a gift from Bob Weinberg (Addgene, Watertown, MA, USA, plasmid #18801) (Onder *et al*., 2008).

### 2.5 Cell proliferation, viability, cell cycle, and migration analysis

Cell proliferation was measured using 3‐(4,5‐dimethylthiazol‐2‐yl)‐2,5‐diphenyl‐tetrazolium bromide (MTT; TC191; Himedia, Mumbai, India) cell proliferation assay. Cell viability was measured using the ATP cell viability assay (6016943, ATPlite Luminescence Assay System, PerkinElmer, Waltham, MA, USA) following the manufacturer's protocol. Cell cycle was determined from propidium iodide assay (PI, TC252; Himedia). Cells were examined in a FACSCalibur flow cytometer (BD Biosciences, San Jose, CA, USA). Cell migration was analyzed by transwell migration assay (CLS3464; Sigma Corning^®^ Costar^®^ Transwell^®^ cell culture inserts; Sigma) and *in vitro* scratch assay (Kramer *et al*., 2013). The width of scratch wounds was measured using TScratch (Geback *et al*., 2009).

### 2.6 Membrane integrity test using lactate dehydrogenase (LDH) assay

After transfection with Cdh23‐siRNA, culture media supernatant was collected for LDH assessment. LDH activity is measured using an LDH cytotoxicity assay (CytoTox 96® Non‐Radioactive Cytotoxicity Assay, Promega, Cat No‐G1780, Madison, WI, USA), according to the manufacturer's protocol. The absorbance is recorded at 490 nm within 20 min after adding Stop Solution with a Microplate Spectrophotometer (Eon Biotek, Winooski, VT, USA). Lysed cells with cell lysis buffer are used as a positive control for total LDH release.

### 2.7 Hanging‐drop assay

Modified hanging‐drop assay was performed as previously described (Redfield *et al*., 1997). Cells were grown, transfected with plasmid/siRNA, incubated for 12 h, dissociated, harvested, counted, and adjusted at a final concentration of 10^6^ cells·mL^−1^. Aliquots of 30 μL were placed on drops and cultured at 37 °C in anchorage‐independent conditions for up to 5 days to assess their ability to form cell aggregates. Photographs were taken at 12 h (24 h after transfection), 72 h, and on the 5th day. To eliminate the artifact of solvent evaporation during the hanging‐drop assay, we maintained a hydrated enclosed chamber. Density and distribution of the cells were measured for a constant length (400–500 nm) using Plot Profile function in imagej (NIH, Bethesda, MD, USA) and plotted as ‘column average plot’, where the *x*‐axis represents the horizontal distance through the selection (diameter) and the *y*‐axis the vertically averaged pixel intensity (gray value). The plot data is used to calculate the area under the curve and the constant diameter compared.

### 2.8 Cloning and expression of Cdh23 plasmid (EC 1–2) containing first two domains

The EC domains EC1–EC2 of mouse Cdh23 (NP_075859) along with the signal peptide was cloned in pcDNA3.1 Mammalian Vector between the *Nhe*I and *Xho*I site using specific forward and reverse primers. The vector backbone was modified with Sortase Recognition Sequence, followed by a GFP tag and a 6×‐His‐Tag placed between the *Xho*I site and *Xba*I site.

The protein expression was done in ExpiCHO Expression System (Thermo Fisher, Cat: A29133) according to the manufacturer's protocol. Briefly, ExpiCHO cells were maintained in ExpiCHO Expression Medium at 37 °C with shaking and were transfected with the Cdh23 (EC1–2) plasmid. The media was taken out after 10 days and dialyzed in HEPES buffer. The dialyzed protein was processed for Ni‐NTA affinity purification. The protein was passed through Ni‐NTA agarose beads (Qiagen, Hilden, Germany) and equilibrated with HEPES buffer. The protein was eluted in the same buffer with an increased concentration of imidazole ranging from 5 to 500 mm (2 mL each). The eluted fractions were run on 12% SDS/PAGE, and western blot was performed using anti‐Cdh23 antibody. The positive protein fractions were concentrated using Amicon Ultra (Merck Millipore, Burlington, MA, USA) and lyophilized. As working stock, 1 μg·μL^‐1^ of Cdh23 and BSA protein in sterile water was used as (10 μL dissolved in 1 mL medium).

### 2.9 Statistical analysis

Uunpaired two‐tailed *t*‐test, non‐parametric Mann–Whitney test, ANOVA, and survival analysis using Log‐rank (Mantel–Cox) test were done with graphpad prism 5 (Graph Pad Software, San Diego, CA, USA). Two‐sided chi‐square/Fisher tests were used to compare the proportion of patients in terms of IHC and TCGA with regard to Cdh23 expression. A *P*‐value of < 0.05 was denoted as a statistically significant difference.

## 3 Results

### 3.1 Cdh23 is uniformly expressed at the cell boundaries in normal tissues and mediates cell–cell adhesion

**Figure 1:**
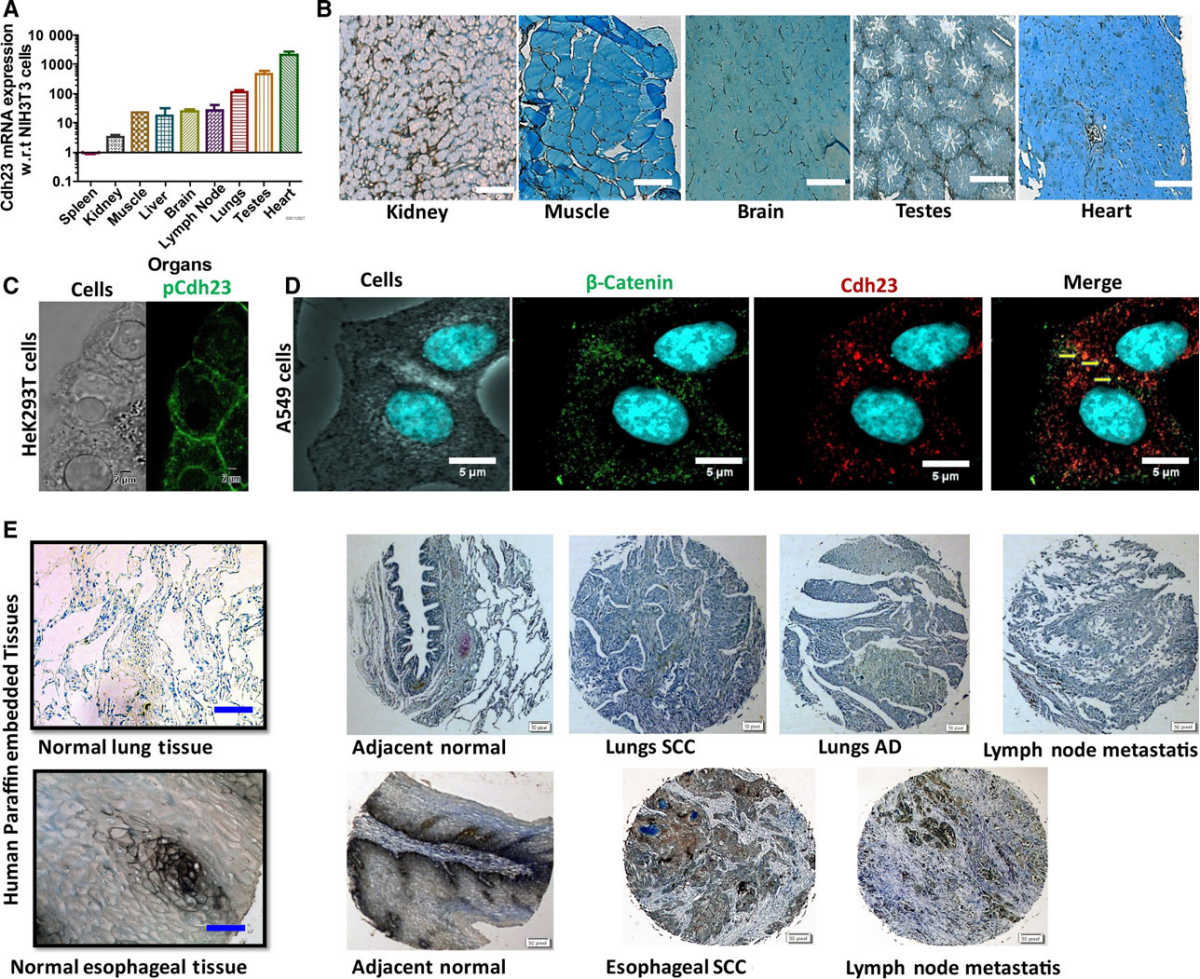
Cdh23 is uniformly expressed at the cell boundaries of various tissues. (A) Cdh23 mRNA expression in various mice tissues was measured by qRT‐PCR. To minimize the contribution of Cdh23 mRNA from fibroblasts, NIH3T3 fibroblast cells were used as a background control, and the relative expression of Cdh23 mRNA (expressed as mean expression ± SEM) for each tissue was obtained after subtracting the background control. (B) Paraffin‐embedded tissue sections of various mice tissues (kidney, muscle, brain, testes, and heart) were IHC‐stained with 3,3′‐diaminobenzidine (DAB) to indicate the expression of the Cdh23 protein (brown color, 20× magnification). Scale bar: 150 μm. (C) Cdh23 was over‐expressed in HEK293T cells (Green) using Cdh23‐GFP plasmid (pCdh23) and the protein expression was recorded by monitoring the GFP‐fluorescence using a confocal microscope (60× magnification). Scale bar:  2 μm. (D) Adenocarcinomic human alveolar basal epithelial cells (A549 cells) expressing intrinsic Cdh23 were immunocytochemically (IC) stained with fluorescent antibodies of β‐catenin with CF594 (green, second panel) and Cdh23 with CF633 (red, third panel). Co‐localization of Cdh23 with β‐catenin (Orange) was marked by yellow arrows (last right panel; 60× magnification). Scale bar: 5 μm). (E) TMA section of Lung (top row) and esophageal cancer (bottom row) were stained by IHC using DAB to show the expression of Cdh23 (brown color; normal tissues in 20× magnification). Scale bar: 150 μm. Carcinoma tissues in 4× magnification. Scale bar: 500 μm.

To verify the diversity in Cdh23 expression experimentally as reported in THPA database (Fig. S2a), we estimated the mRNA expression in several mice tissues (BALB/c mice) and obtained a relatively low expression in spleen and high expression in testes and heart (Fig. 1A). IHC staining marked the expression of Cdh23 protein at the cell boundaries in various normal mice tissues including kidney, muscle, brain, testes, and heart (Fig. 1B). However, the expression patterns was noticeably different from Ecdh and Ncdh when compared simultaneously on similar tissues (Fig. S2b). Cdh23 proteins were also expressed in IHC of human TMA slides of lung cancer (LC) and esophageal squamous cell carcinoma (ESCC) (Fig. S2c) at the cell boundaries in both healthy and cancer tissues (Fig. 1C). With the overexpression of GFP‐labeled Cdh23, we also noticed the homogeneous and continuous distribution of Cdh23 at the cell–cell junctions of HEK293T cells (Fig. 1D). Next, to locate Cdh23 in cells with intrinsic expression of this cadherin, we performed a quantitative qRT‐PCR‐based sorting of cancer cells by Cdh23‐mRNA expression (Cdh23 IS1 primers; Tables S1 and S2). We observed a moderate expression of Cdh23‐mRNA in lung AD (LUAD) A549 cells (Fig. S2d), and performed IF staining of the cells using Cdh23 (EC27‐specific) antibody. We observed distinct puncta at the cell–cell boundaries that were co‐localized with β‐catenin (Fig. 1E), referring to their involvement in cell–cell adhesion.

### 3.2 HEK293T cells are used as model cells to decipher the function of Cdh23‐mediated cell–cell junction

The next aim was to understand the functional role of Cdh23 in normal tissues and what cancer cells gain or lose in regulating the protein. We used HEK293T cells as a model and studied the cellular activities on overexpression/inhibition of Cdh23 protein. HEK293T cells express a low level of both epithelial as well as mesenchymal [cell‐adhesion molecules (CAM)] (Inada *et al*., 2016) and are widely used as a model cell‐line to study membrane proteins (Lin *et al*., 2015). We observed low expression of intrinsic Cdh23 in HEK293T cells at both mRNA and protein level (Fig. S3a). Further, transfection with Cdh23‐siRNA showed a dose‐dependent decrease in Cdh23 mRNA expression (50–200 pmol, 2.5–10 fold) and protein expression (Fig. S3b,c).

#### 3.2.1 Cdh23 silencing did not affect cell proliferation or cell cycle in HEK293T cells

**Figure 2:**
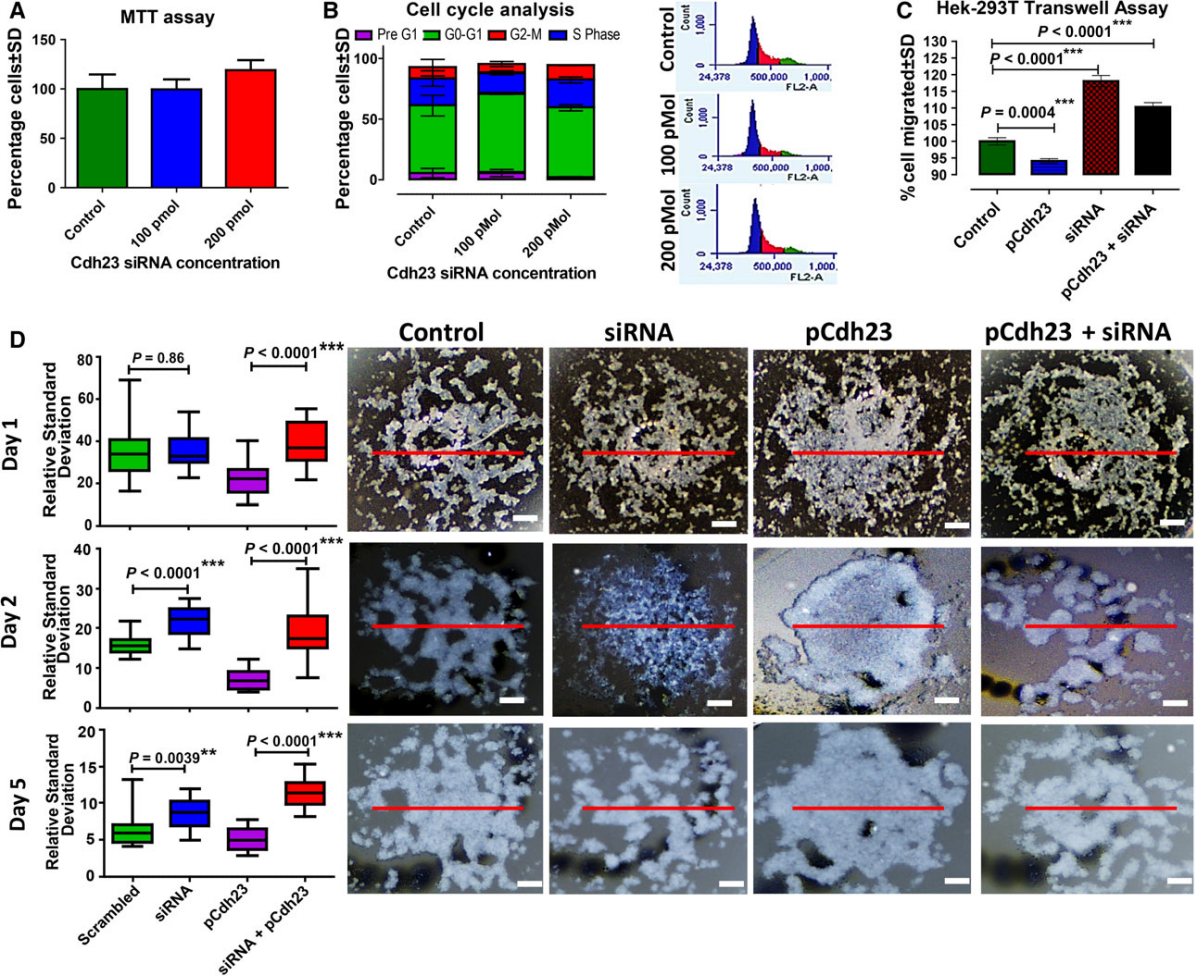
Cdh23 promotes cell migration and aggregation but not cell proliferation. (A) HEK293T cells were transfected with 100 and 200 pmol of Cdh23 siRNA; after 72 h, cell proliferation/viability was measured using MTT assay by monitoring the absorbance at 570/630 nm. The absorbance was then expressed as a percentage of cells in comparison with the control (scrambled siRNA, considered 100%; mean ± SD values for each time point were compared with regard to scrambled control using a two‐tailed paired *t*‐test). (B) Cell cycles were determined by quantitation of DNA content using PI as staining in flow cytometry after 72 h of transfection with 100 and 200 pmol of Cdh23‐siRNA. The data were expressed as a percentage of cells in different cell cycle phases. Mean ± SD values for each time point were compared with regard to scrambled control using a two‐tailed paired *t*‐test. (C) Transwell migration assay, and (D) hanging‐drop assay were performed on scrambled‐transfected HEK‐293T cells (control), Cdh23 over‐expressed HEK293T cells (pCdh23), 200 pmol Cdh23‐siRNA (siRNA)‐transfected HEK‐293T cells, and both Cdh23‐siRNA and pCdh23 (pCdh23 + Cdh23‐siRNA)‐transfected HEK‐293T cells (4× magnification). Scale bar: 1.2 mm. In transwell assay, the data were expressed as the percentage of cells that migrated through the membrane compared with control (100%) on the *y*‐axis and without pCdh23/siRNA on the *x*‐axis. Mean ± SD values were compared with the with regard to scrambled control using a two‐tailed paired *t*‐test. In the hanging‐drop assay, the HEK293T cells were treated as mentioned previously for the migration assay, marked in the figure, the treated cells observed for days, and each picture analyzed in gray scale using the free version of imagej. The change in the density was monitored and the spatial distribution of HEK293T cells with the number of days. To quantify the observed changes in the images, a LoR (red‐line) was plotted, and the RSD (RSD = SD in the intensity/mean‐intensity) along the LoR was estimated (mean ± SEM values with regard to respective controls were compared using two‐tailed Mann–Whitney Test) as shown in the righthand most side panel for day 1, day 3, and day 5 of given treatments (on *x*‐axis). (**P < 0.01, ***P < 0.001).

Inhibiting Cdh23 showed no toxic effect on the cultured cells when analyzed in the presence of 100 and 200 pmol Cdh23‐siRNA. No significant change was observed [percentage of cells ± standard deviation (SD); Fig. 2A] at 100 pmol (99.51 ± 28.65) or 200 pmol (119.1 ± 28.67) of Cdh23‐siRNA in the MTT assay when compared with the scrambled control (100 ± 41.70), indicating the negligible contribution of Cdh23 in cell proliferation. Further analysis in the distribution of cells (percentage of cells ± SD) at different cell cycle phases (Fig. 2B) at 100 pmol (Pre G1, 5.55 ± 3.05; G0–G1, 65.15 ± 0.75; S‐Phase, 17.35 ± 1.85; G2‐M, 7.15 ± 2.15) and 200 pmol (Pre G1, 1.8 ± 0.7; G0–G1, 57.7 ± 2.5; S‐Phase, 22.75 ± 2.65; G2‐M, 9 ± 6.6) of Cdh23‐siRNA in comparison with scrambled control (Pre G1, 5.3 ± 3.8; G0–G1, 55.75 ± 8.35; S‐Phase, 22.45 ± 6.35; G2‐M, 12 ± 0.1) featured no significant differences to indicate any participation of Cdh23 in regulating the cell‐cycles.

#### 3.2.2 Overexpression of Cdh23 stalls cell migration, and inhibition promotes migration

As cell‐adhesion proteins are the primary targets during cell migration, we next focused on the migration of HEK293T cells in the presence and absence of Cdh23. We transfected the cells with a Cdh23‐GFP plasmid for overexpression and subsequently sorted the transfected GFP‐positive HEK293T cells (GFP^+^) from untransfected GFP‐negative cells (GFP^−^) using flow cytometry. We used 5 × 10^4^ cells·mL^−1^ of both cell‐types independently for transwell‐migration and hanging‐drop assays (Fig. S3d).

In transwell migration assays, GFP^+^ HEK293T cells overexpressing Cdh23 showed the low migration of cells (6% lower, number of cells move across the membrane = 148.07 ± 3.97, *P* < 0.05) across the porous membrane towards nutrition‐rich media compared with untransfected GFP^−^ cells (157.35 ± 6.17). To evaluate the cell migration in the absence of Cdh23, both GFP^−^ and GFP^+^ cells were transfected with 200 pmol Cdh23‐siRNA, and noticed significantly higher migration ability of both cells (siRNA‐transfected GFP^+^ cells, 10% higher, 173.57 ± 7.35, *P* < 0.05; and; siRNA‐transfected GFP^−^ cells, 18% higher, 185.78 ± 9.48, *P* < 0.05) than the respective control cells (Fig. 2C).

The involvement of Cdh23 in the cell–cell aggregation was verified using hanging‐drop assay with cell‐lines after silencing Cdh23. Both GFP^−^ and GFP^+^ cells were transfected with 200 pmol of Cdh23‐siRNA, incubated for 12 h, then dissociated in the trypsin‐free buffer, harvested, and finally counted at a final concentration of 10^6^ cells·mL^−1^ for the assay. Twenty drops of 30 μL each were cultured at 37 °C in anchorage‐independent conditions for up to 5 days and periodically photographed the drops from the top‐view after 12 h (Day 1), 72 h (Day 3), and 120 h (Day 5). The analysis of the cell density and cell distribution was done based on gray scale intensity using imagej software, where more gray scale intensity corroborates with a greater density of cells. Distinct differences in the extent of cell aggregation were noticed visually between cells lacking (discrete and small islands of white clumps) and overexpressing Cdh23 (overly spread out, white clumps; Fig. 2D). For quantifying the aggregation, a straight line of reference (LoR) of fixed length (300 nm) was drawn at the center of each drop covering all the cell aggregates (shown as red line, Fig. 2D) and the fluctuations in the intensity obtained (in gray scale) for each pixel (imagej, version 1.51u) along the LoR. The fluctuations (Fig. S3E) measure the extent of aggregation; there are more fluctuations in smaller clumps with less aggregation‐propensity. We represented the intensity fluctuation as the relative standard deviation (RSD = SD*100/mean area) that is necessarily the SD of intensity normalized by the mean intensity. To avoid any artifacts in the analysis, we moved the LoR systematically up and down equal distances for all samples and measured corresponding RSD (expressed as RSD ± SD). Apart from the changes in the intensity‐fluctuation profile, the overall pattern in the RSD values for varying experimental conditions (control, Cdh23‐siRNA, GFP^+^, and siRNA‐GFP^+^) remained the same. At Day 1, no significant difference in RSD was observed between scrambled‐transfected (scrambled, 34.63 ±13.36) and Cdh23‐siRNA‐transfected GFP^−^ cells (34.35 ± 7.96). This is possibly due to low expression of intrinsic Cdh23 in HEK293T cells. However, scrambled‐transfected GFP^+^ cells (21.94 ± 7.9) showed significantly lower RSD (*P* < 0.0001) than Cdh23‐siRNA‐transfected GFP^+^ (38.44 ± 9.5) and GFP^−^ cells (34.63 ± 13.36). Significantly low RSD for scrambled siRNA‐transfected GFP^+^ cells indicate increased aggregation on over‐expression of Cdh23. On Day 3, as the effect of siRNA progresses, we noticed a significant difference (*P* < 0.001) in the scrambled‐transfected (15.98 ± 2.48) and Cdh23‐siRNA‐transfected (siRNA, 21.69 ± 3.72) GFP^−^ cells with lower RSD for the latter, reconfirming that the silencing of the intrinsic Cdh23 inhibits cell aggregation (Fig. 2D). Following a similar trend, the difference in the RSD between scrambled‐ (pCdh23, 7.21 ± 2.59) and Cdh23‐siRNA‐transfected (pCdh23 + siRNA, 19.13 ± 6.43) GFP^+^ cells was significantly increased (*P* < 0.01), further evidence that lack of Cdh23 resulted in decreased aggregation of cells (Fig. 2D). By Day 5, we noticed a decrease in the effect of siRNA and thus reduction in the differences in RSD (scrambled, 6.44 ± 2.3; siRNA, 11.05 ± 2.02; and pCdh23, 5.09 ± 1.5; pCdh23 + siRNA 11.05 ± 2.02). Overall, the transwell assay and hanging‐drop assay jointly indicated that silencing of Cdh23 expression resulted in decreased cell aggregation and enhanced cell migration.

### 3.3 Cdh23 is a suppressor of cancer metastasis: studies on normal and cancer patients

#### 3.3.1 The decrease in Cdh23 mRNA expression promotes cancer metastasis: from *in silico* analysis

Since the disintegration of cell–cell adhesion from primary tissues (Steeg, 2016) and acceleration in cell migration are significant steps in metastatic dissemination, we traced the relation between Cdh23 expression and cancer metastasis. We focused on two cancer types, LUAD and ESCC from TCGA cbioportal (http://www.cbioportal.org/; Data S1), as TCGA and existing literature (Sawada *et al*., 2016; Seo *et al*., 2012) reported a significant reduction of Cdh23 expression in these two cancers for their respective patient cohort. We divided individual samples into two groups based on their mean Cdh23 mRNA expression quantified as RNA‐Seq by expectation maximization (RSEM) values. Those with higher than the mean expression are named ‘High‐group’ and and those with lower than the mean expression ‘Low‐group’. To determine survival probability, both groups were compared based on the hazard ratio (HR) using the Log‐rank (Mantel–Cox) test (Spruance *et al*., 2004). HR is the ratio between the risk of the outcome in High‐group and risk of the outcome in the Low‐group. The risk is estimated for patients who survived for > 2 months (60 days) in order to exclude those who registered their disease at their terminal stages. According to the test, an HR value < 1 indicates that the High‐group has better survival than the Low‐group.

**Figure 3:**
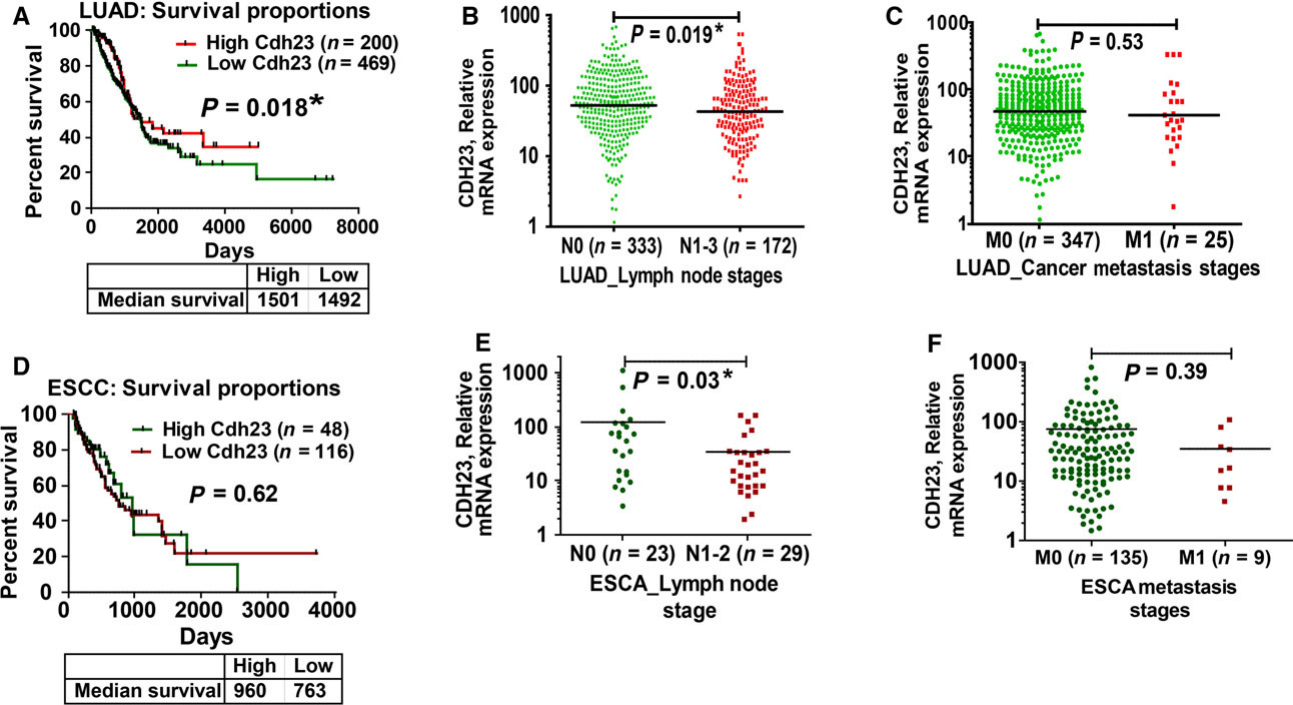
Cdh23 is uniformly expressed in normal tissues and down‐regulated in cancer. (A) Based on mean Cdh23 RSEM values (RE = 78.31), patients in LUAD were segregated into two groups, high‐expression of Cdh23 (red) and low‐expression of Cdh23 (green), and their survival in days was plotted. The survival proportion for those who survive for more than 2 months having high (no. of patients, *n* = 200) and low (*n* = 469) expression was compared using the Log‐rank (Mantel–Cox) test. (B) Patients with advanced lymph node status (N1–2, *n* = 172, RE = 70.26, green) were compared with N0 stage (*n* = 333, RE = 84.78, red). (C) Patients with metastasis (M1, *n* = 25, RE = 42.10, green) were compared with M0 stage (*n* = 347, RE = 47.30, red). Similar to LUAD, patients suffering from ESCC were segregated into two groups based on the mean Cdh23 RSEM values (RE = 63.06), high (red) and low (green) expression, and their survival was plotted. (D) The survival proportion for those with survival days > 60 having high (*n* = 48, red) and low (*n* = 116, green) Cdh23 expression was compared using Log‐rank (Mantel–Cox) test. (E) Patients with advanced lymph node status (N1–2, *n* = 29, RE = 34.69, green) were compared with N0 stage (*n* = 23, RE = 123.4, red). (F) Patients with metastasis (M1, *n* = 9, RE = 34.87, green) were compared with M0 stage (*n* = 135, RE = 74.67, red). (*P < 0.05).

In LUAD (*n* = 669), High‐group [*n* = 200, mean relative expression (MRE) of Cdh23‐mRNA > 78.31, median survival = 1501 days] has better survival [Fig. 3A, HR = 0.72, 95% confidence interval (CI) = 0.55–0.95, *P* = 0.018] than the Low‐group (*n* = 469, MRE < 78.31, median survival = 1492 days). Further, based on lymph node status, patients in N0 stage with no observable lymph node involvement (MRE = 84.78, *n* = 333) have significantly higher Cdh23 mRNA expression (Fig. 3B) compared with those with advanced N stages (N1–3, *P* = 0.019, MRE = 70.26, *n* = 172). Moreover, patients with no metastasis, M0 stage (MRE = 47.30, *n* = 347) have higher Cdh23 expression (Fig. 3C) compared with those with metastasis (M1, MRE = 42.10, *n* = 25, *P* = 0.53).

Similarly, in ESCC (*n* = 164), patients in the High‐group (*n* = 48, MRE > 63.06, median survival = 960 days) have better survival (Fig. 3D, HR = 0.87, 95% CI = 0.52–1.4, *P* = 0.61) than those in the Low‐group (*n* = 116, MRE < 63.06, median survival = 763 days). Based on lymph node status, patients in N0 stage with no observable lymph node involvement (MRE = 123.4, *n* = 23) have significantly higher Cdh23 mRNA expression (Fig. 3E) compared with those with advanced N stages (N1–2, *P* = 0.03, MRE = 34.69, *n* = 29). Moreover, patients with no metastasis, M0 stage (MRE = 74.67, *n* = 135) have higher Cdh23 expression (Fig. 3F) than those with metastasis (M1, *P* = 0.39, MRE = 34.87, *n* = 9).

Interestingly, independent analysis of the TCGA data by THPA also revealed the strong connection of cancer metastasis and patient survival with Cdh23 expression (Table S3) in cervical cancer (CESC), uterine cancer (UCEC), and skin cancer (SKCM), in addition to LUAD and ESCC.

#### 3.3.2 Tissue microarray analysis (TMA) corroborates the decrease in Cdh23 expression with metastasis

To relate the protein expression to metastasis, we performed IHC staining of the TMA slides for LCTA, ESCC, and metastasis array (ESTA; Fig. S2c). Since the TMA slides contain multiple micro‐dissected tissues including adjacent lung tissue, malignant tissue from the primary tumor, and lymph node metastatic tissue from the same patient, we referred to them together as tissue samples. The staining intensity and the extent of staining were analyzed using IHC profiler in imagej (Varghese *et al*., 2014). The mean intensity of the healthy tissue was used as a limit to segregate samples into two groups: higher than the mean as high‐Cdh23 and lower than the mean as low‐Cdh23. Next, we estimated the number of tissues falling into each of the groups based on staining intensity and correlated them with the different stages of the tumor (Table 1).

**Table 1:** Frequency of patients having low and high Cdh23 expression for different stages of cancer (in percentage) in LC and esophageal cancer tissue array (TMA) and TCGA patient groups (LUAD, LUSC, ESCC)

		Low Cdh23 (n%)	High Cdh23 (n%)	*P* value
Cdh23 protein expression (IHC staining, *n* = 92)				
LCTA (TMA)	Healthy lung tissue	4 (50)	4 (50)	1
	Adjacent lung tissue	26 (68.4)	12 (31.6)	0.27
	SCC	10 (66.6)	5 (33.4)	0.36
	AD	11 (73.3)	4 (26.7)	0.25
	Large cell carcinoma	5 (62.5)	3 (37.5)	0.5
	LN_Metastasis SCC	2 (100)	0 (0)	0.33
	LN_Metastasis AD	5 (83.3)	1 (16.7)	0.23
LC Cdh23 mRNA expression (TCGA cbioportal)				
LUAD	Mean mRNA expression (RNA Seq V2 RSEM) = 78.31			
	Lymph node stage (American Joint Committee on Cancer Code, *n* = 504)			
	N0	217 (65.2)	116 (34.8)	1
	N1	69 (72.6)	26 (27.4)	0.10
	N2–3	56 (73.7)	20 (26.3)	0.09
	Metastasis (American Joint Committee on Cancer Metastasis Stage Code, *n* = 372)			
	M0	238 (68.6)	109 (31.4)	1
	M1	18 (72)	7 (28)	0.45
LUSC	Mean mRNA expression (RNASeq V2 RSEM) = 143.87			
	Lymph node stage (American Joint Committee on Cancer Code, *n* = 495)			
	N0	240 (75.2)	79 (24.8)	1
	N1	100 (76.3)	31 (23.7)	0.45
	N2–3	42 (93.3)	3 (6.7)	0.002^b^
	Metastasis (American Joint Committee on Cancer Metastasis Stage Code, *n* = 418)			
	M0	312 (75.9)	99 (24.1)	1
	M1	5 (71.4)	2 (28.6)	0.53
Cdh23 protein expression (IHC staining, *n* = 192)				
Esophagus SCC tissue array (TMA)	Cancer adjacent normal esophageal mucosa	5 (50)	5 (50)	1
	Cancer adjacent tissue	23 (85.2)	4 (14.8)	0.04^a^
	SCC	85 (72.6)	32 (27.4)	0.12
	Metastatic SCC	32 (84.2)	6 (15.8)	0.035^a^
Esophageal cancer Cdh23 mRNA expression (TCGA cbioportal)				
ESCC	Mean mRNA expression (RNASeq V2 RSEM) = 63.06			
	Lymph Node Stage (American Joint Committee on Cancer Code, *n* = 52)			
	N0	16 (69.6)	7 (30.4)	1
	N1	24 (92.3)	2 (7.7)	0.04^a^
	N2–3	1 (33.3)	2 (66.7)	0.27
	Metastasis (American Joint Committee on Cancer Metastasis Stage Code, *n* = 144)			
	M0	101 (74.8)	34 (25.2)	1
	M1	7 (77.8)	2 (22.2)	0.60

a *P* < 0.05.

b *P* < 0.01.

In the case of LCTA, we observed a higher population of tissues (12–23% more) that are adjacent to primary tumor (adjacent lung tissue, 68.4%) and malignant (62.5–73.3%), falling in the low‐Cdh23 group in comparison with normal lung tissue (50%). Even more interesting, in LCTA, a higher percentage of patients with lymph node metastatic tissues (83–100%) showed lower expression of Cdh23 than lung SCC (LUSC; 66.6%) and AD (73.3%). Thus patients with LC progression showed low expression of Cdh23. Similarly, in ESTA, metastatic SCC (84.2%) has a higher percentage of tissues with low‐Cdh23 expression compared with the SCC (72.6%) and normal esophageal mucosa adjacent to cancer (50%), further strengthening our observation of a decrease in Cdh23 expression during ESTA progression.

Similar to our experimental observations, TCGA has also reported a higher percentage of patients with advanced lymph node status [> N1, LUAD, 74%; LUSC, 93%; ESCC (N1), 92%] and metastasis stage (M1, LUAD, 72%; LUSC, 71%, ESCC, 78%) in the low‐Cdh23 expression group. This inference further supports the promotion of metastasis by the down‐regulation of Cdh23 expression (Table 1). Overall, *in silico* analysis and TMA analysis suggest that Cdh23 is decreased in cancer, which is further decreased in advance lymph node stages and metastatic stages, suggesting Cdh23 suppresses cancer cell metastasis.

#### 3.3.3 Promoter methylation is responsible for down‐regulation of Cdh23 in cancer

**Figure 4:**
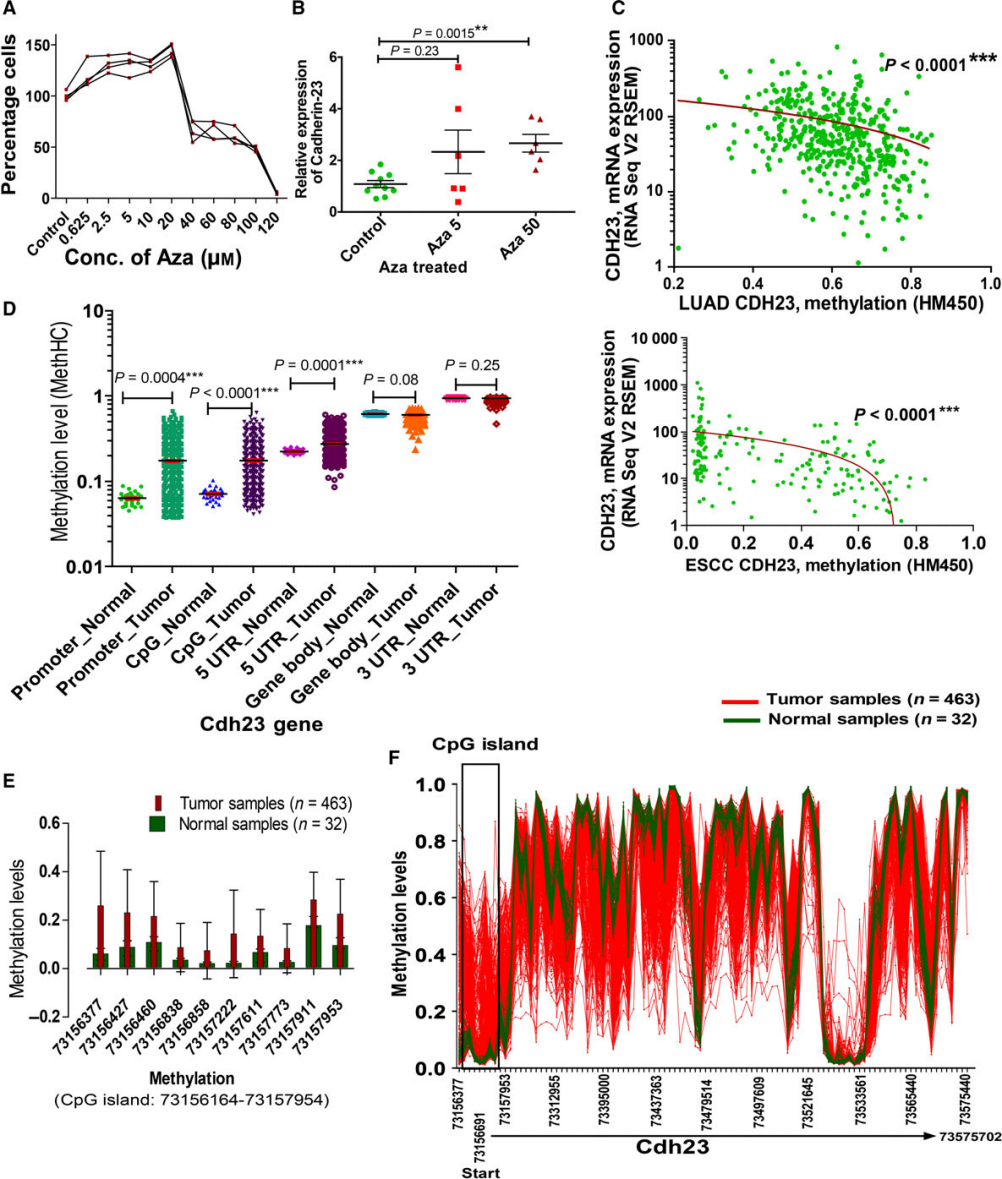
Promoter methylation regulates Cdh23 expression. (A) Dose‐response curves (four repeats) by MTT assay showing A549 cell viability with increasing concentrations of Aza with regard to 100% viability with no drug (control). (B) Relative expression of Cdh23 mRNA was measured by qRT‐PCR after treating with 5 and 50 μm of Aza (mean ± SD values were compared with control using two‐tailed paired *t* test. (C) Expression of Cdh23 in patients (*n* = 456, RSEM values) was correlated to methylation levels (TCGA, HM450) for LUAD (Spearman *r* = −0.360, 95% CI −0.4396 to −0.2749) and ESCC (Spearman *r* = −0.4586, 95% CI −0.5684 to −0.3327). (D) Comparison of relative methylation levels in Cdh23 gene at promoter, CpG region, 5′‐UTR, gene body and 3′‐UTR for LUAD tumor (*n* = 463) and normal (*n* = 32) samples as obtained in MethHC database (mean ± SD values were compared using the two‐tailed Mann–Whitney test). (E) Relative methylation levels (mean ± SD) as reported in TCGA Wanderer database at specific CpG island (Chromosome 10: 73156164–73157954) near the Cdh23 promoter for LUAD tumor (*n* = 463) and normal (*n* = 32) samples. and (F) Pattern of relative methylation levels in Cdh23 gene (Chromosome 10: 73156377–73575440) in normal (*n* = 32, green) and tumor (*n* = 463, red) samples. (**P < 0.01, ***P < 0.001).

To decipher the molecular mechanism of the down‐regulation, we treated A549 cells with various small molecule epigenetic modulators targeting DNA methylation (5‐Aza‐2′‐deoxycytidine, AZA; Fig. 4A) and histone modifications (histone deacetylation inhibitor, trichostatin A, sodium butyrate, and valproic acid; Fig. S4a–j). We first generated the dose‐response curve for each inhibitor and treated the A549 cells with the optimized doses (Fig. S4a–j). Since inhibition of DNA methylation and histone deacetylation should result in recovery of Cdh23 mRNA expression, qRT‐PCR was performed to analyze the expression of Cdh23 mRNA after the treatments. Only Aza was able to recover Cdh23 expression (Fig. 4B) at a dose concentration of 5 μm (2.32 ± 2.06‐fold, *P* = 0.23) and 50 μm (2.6 ± 0.8, *P* = 0.0015), implicating DNA‐methylation as the prime suspect for regulating Cdh23 expression.

Next we correlated the extent of DNA methylation with mRNA expression for Cdh23 from the TCGA and noticed a negative correlation in both LUAD (Fig. 4C, *n* = 456, Spearman *r* = −0.36, 95% CI −0.43 to −0.27, *P* < 0.0001) and ESCC (Fig. 4C, *n* = 185, Spearman *r* = −0.46, 95% CI −0.57 to −0.33, *P* < 0.0001). To verify how methylation can control the Cdh23‐expression, we compared the methylation sites in various regions of Cdh23 gene (Promoter, CpG island, 5′UTR, gene body, 3′‐UTR; Fig. S4a) for normal (*n* = 32) and LUAD tumor (*n* = 463) patients as deposited in MethHC database in TCGA. We observed significantly higher methylation at the promoter‐region (~ 2 kb from TSS; Fig. S4a), CpG island, and 5′‐UTR (*P* < 0.001) in tumor than in normal cases (Fig. 4D). Further, zooming the promoter‐region near the ‘start’ site of Cdh23 at chromosome 10 (73156164–73157954), we observed a CpG island (73156164–73575440) which showed higher methylation for LUAD tumor (*n* = 463, red) samples than the normal (*n* = 32, green) cases (Fig. 4E,F; http://maplab.imppc.org/wanderer/; Data S1). We also noticed a strong correlation of increased promoter methylation with decreased Cdh23 expression in most of the solid cancers (from MethHC database; Fig. S4b; Huang *et al*., 2015). Further, we observed increased methylation for both LUAD and ESCC (Fig. S4c,d) with higher lymph node status (N1–2) than no lymph node involvement (N0), reinforcing the strong correlation of cancer metastasis with the down‐regulation of Cdh23 mRNA.

#### 3.3.4 Inhibition of Cdh23‐mediated cell–cell contacts accelerates cell migration: studies on cancer cell‐lines

**Figure 5:**
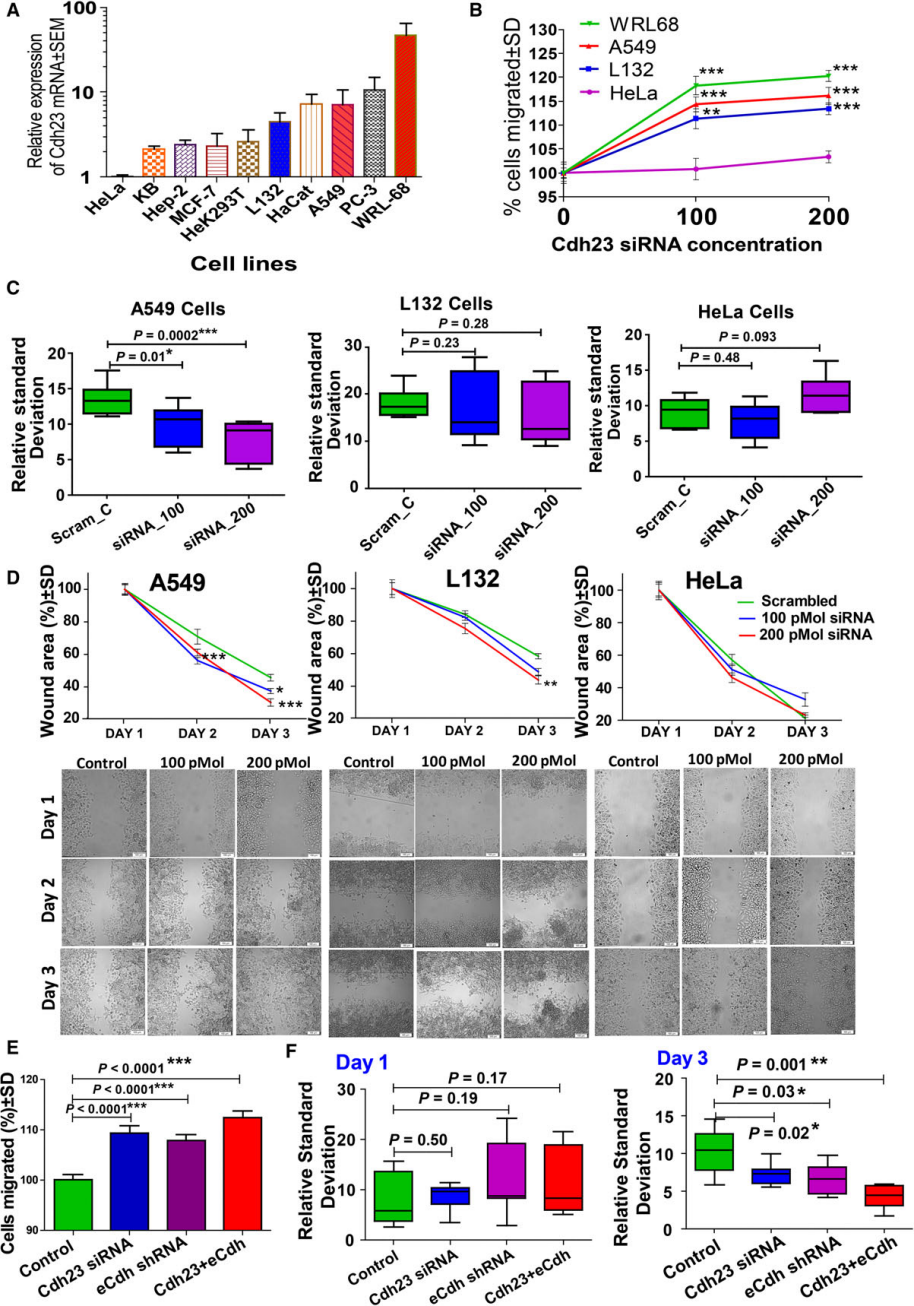
Silencing Cdh23 promotes cancer cell migration and inhibits cell aggregation. (A) Relative expression of Cdh23 mRNA (expressed as mean ± SEM) was estimated in various cancer cell lines by qRT‐PCR and plotted as a histogram. Based on their intrinsic expression of Cdh23, four cell‐lines, HeLa, L132, A549, and WRL68, HeLa being smallest and WRL68 the biggest, were sorted for the migration and aggregation assays. (B) Transwell cell migration assay was performed with the four sorted cell‐lines, HeLa, L132, A549, and WRL68, after treating with various doses of Cdh23‐siRNA and plotted as a percentage of cells (mean ± SD values were compared with regard to scrambled control using a two‐tailed paired *t*‐test) which migrated through the membrane. (C) Modified hanging‐drop assay was performed as previously with the four sorted cell‐lines 72 h after the treatment with three different doses of Cdh23‐siRNA (scrambled as control, 100, and 200 pmol). The quantitative estimation of the density and the distribution of cells on the spot area were measured by following the previously described method and plotted RSD (mean ± SEM values were compared with regard to scrambled control two‐tailed Mann–Whitney test) for individual cell lines. (D) Wound‐healing assay was performed after 12 h of transfection of scrambled control (Scram_C) and Cdh23‐siRNA (100 pmol, siRNA_100; 200 pmol, siRNA_200) to three cell‐lines (HeLa, L132, and A549) and the percentage decrease in the free space of wound area was calculated using T‐scratch (on the *y*‐axis) for Day 1, Day 2, and Day 3 (days on *x*‐axis; mean ± SD values were compared with regard to scrambled control for each time point using a two‐tailed paired *t*‐test, 10× magnification). Scale bar: 100 μm. (E) The transwell cell migration assay was performed for three cell‐lines (HeLa, L132, and A549) after treating with 200 pmol scrambled control (Control), 200 pmol Cdh23‐siRNA, 2 ng ECdh‐shRNA, and combination of Cdh23‐siRNA + ECdh‐shRNA, and expressed as percentage of cells migrated through the membrane compared with control (100%; mean ± SD values were compared with regard to control using a two‐tailed paired *t*‐test). (F) The hanging‐drop assay was repeated with A549 cells to compare the contribution of ECdh in aggregation. The cells were transfected with 200 pmol scrambled control (Control), 200 pmol Cdh23‐siRNA, 2 ng ECdh‐shRNA, and a combination of Cdh23‐siRNA + ECdh‐shRNA, and analyzed as before from the density and the distribution of cells on the drop‐spot. RSD values (mean ± SEM values were compared using two‐tailed Mann–Whitney Test) were plotted for Day 1 (24 h) and Day 3 (72 h) for different treatment conditions. *P < 0.05, **P < 0.01, ***P < 0.001.

Next, we experimentally verified the function of intrinsic Cdh23 in cancer cell‐lines differentially expressing Cdh23. To sort the cell‐lines, we experimentally measured Cdh23‐mRNA expression using qRT‐PCR (Fig. 5A). With respect to HeLa (cervical, 1 ± 0.11), expressions in other cell‐lines are: KB (mouth, 2.15 ± 0.33) < Hep2 (cervix, 2.39 ± 0.69) < MCF7 (breast, 2.31 ± 1.89) < Hek293T (kidney, 2.6 ± 2.02) < L132 (lungs, 4.51 ± 2.32) < HaCat (skin, 7.25 ± 2.43) < A549 (lungs, 7.13 ± 6.94) < PC3 (prostate, 10.61 ± 8.38) < WRL68 (embryonic liver, 47.02 ± 37.92). We then selected HeLa, L132, A549, and WRL68 in ascending order of Cdh23 mRNA expression for subsequent experiments.

To determine the role of Cdh23 in cell proliferation and viability, we independently transfected the cells (HeLa, L132, A549, and WRL68) with 100 and 200 pmol Cdh23‐siRNA, and monitored them for MTT cell proliferation assay, ATP cell viability assay, pI cell‐cycle analysis, and LDH assay for membrane toxicity. As expected from previous studies with model HEK293T cells, we noticed no contribution of Cdh23 (Fig. S6).

To evaluate the role of Cdh23 in cell migration, we transfected the cell lines (HeLa, L132, A549, and WRL68) with 100 and 200 pmol Cdh23‐siRNA, and seeded 1 × 10^4^ cells for the transwell migration assays with respective controls (scrambled siRNA‐transfected cells). HeLa cells with the least expression of Cdh23 showed no significant change in the migration at 100 pmol (150.6 ± 10.03) or 200 pmol (154.4 ± 5.42) of Cdh23‐siRNA compared with scrambled control (149.4 ± 4.86). However, L132 (100 pmol, 170.2 ± 9.4; 200 pmol, 173.4 ± 5.71), A549 (100 pmol, 173 ± 7.16; 200 pmol, 175.7 ± 7.95), and WRL68 (100 pmol, 160.1 ± 7.93; 200 pmol 162.8 ± 4.72) showed significant differences (Fig. 5B) with respect to scrambled controls (L132, 152.9 ± 8.13; A549, 151.3 ± 4.8; WRL68, 135.4 ± 8.75), indicating increased cell migration with the silencing of Cdh23.

We performed the hanging‐drop assay to measure the cell aggregation without silencing of Cdh23 and as described previously for HEK293T cells, monitored RSD for quantification (Fig. 5C). We excluded WRL‐68 cells for the assay, as these cells form discrete clumps independently when cultured in a growth medium (Gutierrez‐Ruiz *et al*., 1994). As expected, no significant change in RSD was observed for HeLa cells when treated with 100 pmol (7.86 ± 2.59, *P* = 0.48) or 200 pmol (11.64 ± 2.72, *P* = 0.093) of Cdh23‐siRNA compared with scrambled control (9.11 ± 2.10). L132 showed a decrease in RSD at both 100 pmol (16.62 ± 7.1, *P* = 0.28) and 200 pmol (15.50 ± 6.29, *P* = 0.23) compared with scrambled control (18.29 ± 3.15). A549, which has a higher Cdh23 expression than both the former cell lines, showed a significant decrease in cell aggregation at both 100 pmol (9.78 ± 2.84, *P* < 0.05) and 200 pmol (7.73 ± 2.85, *P* < 0.001) compared with scrambled control (13.57 ± 2.19). Thus, siRNA‐mediated inhibition of Cdh23 showed less aggregation, suggesting its involvement in cell–cell adhesion (Fig. S7a,b,c).

Next, we performed the wound‐healing assay. The three cell lines HeLa, L132, and A549 were grown to 70–80% confluency and transfected with 100 or 200 pmol of Cdh23‐siRNA. After the cells became confluent (24 h), the wound‐healing assay was initiated by a scratch with 10 μL tips. Photographs (*n* = 15 each case) were taken on Day 1 (12 h after transfection), Day 2 (48 h), and Day 3 (72 h) and analyzed by TScratch (Geback *et al*., 2009) (Fig. 5D). HeLa showed no change in the rate of healing with the silencing of Cdh23. However, for L132 and A549, the rate of healing increased significantly with the increase in Cdh23‐siRNA; for A549, there was a significant gap‐closure on Day 3 for the Cdh23‐siRNA sample treated with 200 pmol. Thus silencing of Cdh23 expression also results in a potentially increased cell migration.

Ecdh is a well‐known cell–CAM and its involvement in cell migration is well‐established. We, therefore, considered the Ecdh the gold standard and compared the significance of the changes in cell migration for Cdh23 with Ecdh. Among the three selected cancer cells lines, we quantified from mRNA expression that both Cdh23 and Ecdh are uniformly expressed in A549 (Fig. S7d,e). Thereafter we studied the cell migration by transwell assay and cell aggregation by hanging‐drop assay using A549 cells after transfecting the cells with 200 pmol of Cdh23‐siRNA and 2 ng of Ecdh‐shRNA. Compared with control (166.86 ± 6.86), both Cdh23 (182.47 ± 8.6) and Ecdh (180 ± 6.8) silencing showed higher cell migration (Fig. 5E) to an equal extent, which was further enhanced when both were silenced together (187.47 ± 8.45). Similarly, RSD from hanging‐drop assay showed no significant difference after Day 1 of silencing (Control, 7.83 ± 4.85; Cdh23‐siRNA, 8.80 ± 2.37; Ecdh‐shRNA, 12.15 ± 6.94; Cdh23 + Ecdh, 11.05 ± 6.56). However, on Day 3, compared with control (10.27 ± 2.85), transfected cells, i.e. cells with Cdh23‐siRNA (7.11 ± 1.39) and Ecdh‐shRNA (6.52 ± 1.96), showed a significant decrease in cell aggregation (Fig. 5F), and the decrease was even greater when the proteins were silenced simultaneously (4.23 ± 1.45).

Although silencing of Cdh23 in cancer cells showed no effect on the canonical β‐catenin signal pathway, suggesting that its prevailing function was cell adhesion (Fig. S8a), comparison of various downstream effector targets obtained from the online algorithms biogrid 3.4, Mentha and String analysis showed an anti‐correlation between Cdh23 expression with the expression of many downstream oncogenes and metastasis promoters (Fig. S8b,c).

Overall, *in vitro* analysis of cancer cell lines suggests that Cdh23 can suppress cancer cell migration and promote aggregation. The effect is synergistic to Ecdh and significant in cells where they are uniformly expressed, conforming to their role as a cell–cell adhesion protein and regulating cell migration.

### 3.4 Cdh23 expresses various excretory isoforms that accelerate cell migration

**Figure 6:**
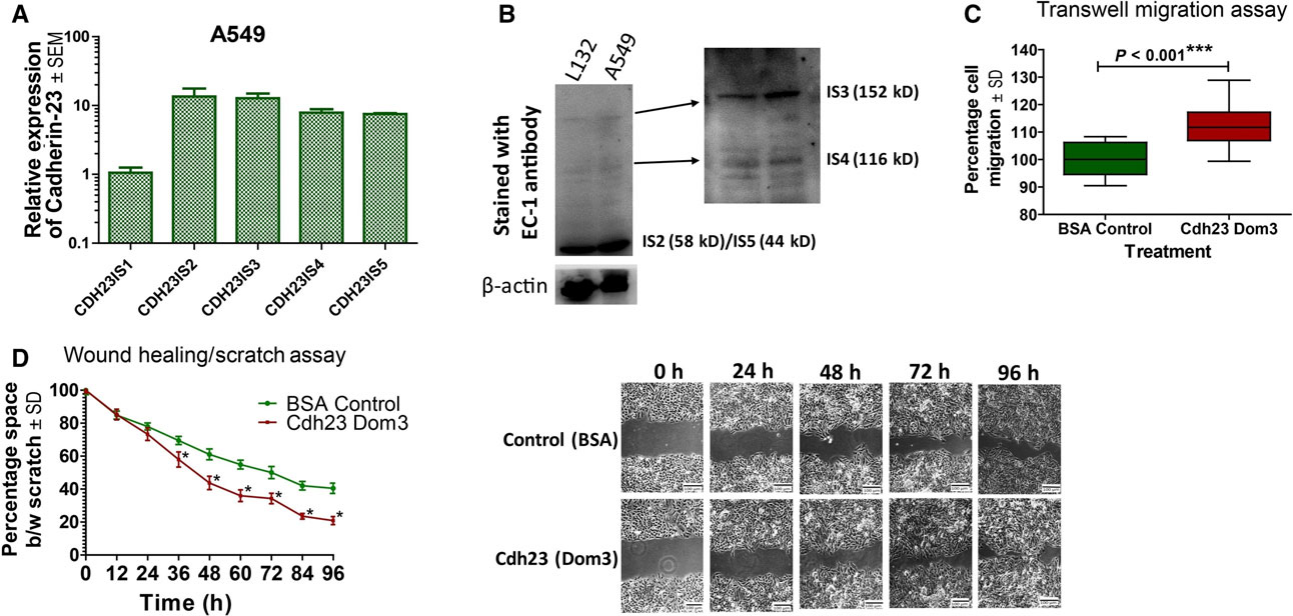
Soluble isoforms of Cdh23 are expressed in A549 cells and regulate cell migration. (A) Relative mRNA expression (mean ± SEM) of various soluble isoforms of Cdh23 (Cdh23 IS1–5) estimated by qRT‐PCR. (B) Western blot (in a 10% SDS gel) of different Cdh23 isoforms (Cdh23 IS1–5) as obtained in the culture media. (C) Transwell migration assay. (D) Wound healing/Scratch assay (10× magnification; scale bar: 100 μm) of A549 cells was monitored in the presence of BSA protein (10 μg·mL^−1^) as control and Cdh23 EC1–2 protein (10 μg·mL^−1^) in the culture media (mean ± SD values for each time point were compared with regard to BSA control using a two‐tailed paired *t*‐test for both transwell migration and (d) wound healing/Scratch assay). (*P < 0.05, ***P < 0.001).

Besides the full‐length Cdh23 isoform (Cdh23 IS1, MW = 370 kD), various other isoforms of Cdh23 with shorter EC domains exist: (1) without transmembrane and cytosolic domains (excreted in the EC matrix as free proteins): isoform 2 (IS2, EC 1–5, MW = 58 kD), isoform 3 (IS3, EC 1–13, MW = 152 kD), isoform 4 (IS4, EC 1–10, MW = 116 kD), isoform 5 (IS5, EC 1–3, MW = 44 kD); (2) with transmembrane and cytosolic isoforms (anchored to membrane): isoform 6 (IS6, EC 21–27, MW = 123 kD), isoform 7 (IS7, EC 21–27, MW = 119 kD); and (3) without any EC domains: isoforms 8 and 9 (only transmembrane and cytosolic domain, MW ~ 27 kD). We noticed a predominant expression of IS2 and IS5 in both protein and mRNA forms, in various cancer cell lines including A549 cells (Figs 6A,B and S9a–j). Expression of IS2 and IS5 was also reported previously for MCF7 cells (Apostolopoulou and Ligon, 2012). Western blot with a Cdh23 EC1‐specific antibody also identified the secretion of IS2/5 in the media (Fig. S9d). Silencing Cdh23 with siRNA, targeting IS1–5, showed a reduced expression of these isoforms (Fig. S9k). To verify whether these are the splice variants of Cdh23, we treated A549 cells with HAT activator (100 μm CTBP, an activator of p300 HAT) and RNA splicing inhibitors (10 μm isoginkgetin). Both the treatments showed an increase in the expression of Cdh23 IS1 (Fig. S9j–k) and a decrease in other isoforms (Fig. S9l) in qRT‐PCR.

Since Cdh23 is known to engage in *trans*‐homodimers at the cell–cell junctions using EC1–2 domains alone Elledge *et al*., 2010, we hypothesized that the excretory isoforms could interact with the EC1–2 domains of Cdh23 IS1 and inhibit the intercellular homodimer interaction between IS1 from the opposing cell surfaces by competitive binding. Loosening of the cell–cell junction by excretory isoforms may facilitates faster cell migration. To validate our hypothesis, we added 10 μg of recombinant Cdh23 EC1–2 in the media and observed a significant alteration in cell migration. Transwell migration assay showed 12.5% increased cell migration (Fig. 5C) in comparison with BSA (10 μg) as a control. Scratch assay/wound‐healing assay with A549 cells in the presence of Cdh23 EC1–2 (10 μg·day^−1^) also demonstrated an increased rate of migration via a faster rate of gap closure than the BSA‐treated (10 μg·day^−1^) A549 cells (Fig. 5D), indicating the significant role of the excretory isoforms in facilitating cell migration in cancer.

## 4 Discussion

*In vitro* studies suggested Cdh23‐mediated strong cell–cell adhesion (Apostolopoulou and Ligon, 2012; Siemens *et al*., 2004). However, biological relevance was lacking. We identified a uniform expression of Cdh23 at the cell–cell boundaries in different normal and cancer tissues (Fig. 1). The Human Proteome Atlas (THPA) also featured the uniform expression of Cdh23 in organs such as brain, lymph node, gastrointestinal tract, testis, and skin (Figs S1 and S3). Similar observations were made in mice (Palma *et al*., 2001). Using HEK293T cells expressing GFP‐labeled Cdh23, we demonstrated the formation of a homogeneous and continuous cell–cell junction by Cdh23 and its co‐localization with β‐catenin. Immunofluorescent staining of intrinsic expression of Cdh23 showed puncta at the homotypic cell–cell junctions of A549 cancer cells (Fig. 1). In summary, the present data indicate the involvement of Cdh23 in cell–cell adhesion.

Cell‐adhesion molecules and their junctional complexes regulate the migration of cells. We, therefore, probed the involvement of Cdh23‐mediated cell–cell junctions in cell aggregation. Overexpression of Cdh23 resulted in reduced cell migration and extended cell–cell aggregation, whereas silencing Cdh23 increased the migration ability of cells in transwell assay, and dislodged the cells into multiple small clusters in the hanging‐drop experiment. These observations not only support the existence of Cdh23‐mediated strong cell–cell junctions but also indicate that Cdh23 is a potential suppressor of migration (Figs 2 and 5).

Extensive data‐mining from THPA and TCGA disclosed the down‐regulation of Cdh23 mRNA in most solid cancers (Fig. 3), including LUAD and ESCC. Further analysis of Cdh23 mRNA expression in LUAD and ESCC revealed a strong correlation of high Cdh23 expression with longer patient survival, whereas patients with advanced lymph node status (N1–2) and metastasis stage (M1–2) showed comparatively low Cdh23 mRNA expression. Our immunohistochemical studies with TMA slides of lung and esophageal cancer also corroborated the TCGA mRNA expression with the Cdh23‐protein expression. We observed less expression of Cdh23 protein in cancer‐adjacent TMA and cancer TMA than normal, and a further decrease in protein expression for metastatic lymph node TMA (Table 1). From our experimental observations with TCGA and THPA we propose an essential role of Cdh23‐mediated strong cell–cell adhesion, which is significantly regulated in cancer.

Dislodgement of the cell–cell association from the primary tumors and migration to a secondary site are critical processes in cancer metastasis. CAM with a strong aggregation index will suppress such processes. The down‐regulation of such CAM favors cell‐migration, thus promoting cancer metastasis (Chen *et al*., 2010). CAM are frequently regulated in cancer, i.e. by down‐regulating the constituent protein molecules by epigenetic mechanisms (Cavallaro and Christofori, 2004), mutations (Jeanes *et al*., 2008), and switching the molecule with other family members (Lamouille *et al*., 2014) or isoforms (Liao *et al*., 2013; Matos *et al*., 2017). In the search for possible factors triggering the down‐regulation of Cdh23 expression, we noted an anti‐correlation of Cdh23‐expression with DNA‐methylation (Fig. 4), whereas in patients with low expression (N1–2, and M1–2 stage patients) DNA methylation was comparatively higher. In support of our observations, a genome‐wide analysis also reported a significant correlation between CpG methylation and Cdh23 expression (Zhu *et al*., 2016). We observed the expression of various isoforms without the transmembrane domain (isoforms 2–5) which were predominantly expressed in many cancer cell lines. Since Cdh23 forms homodimers with the first two domains, these soluble isoforms (with EC1–2 domain) can interfere with such homodimer formation between cells. We also observed that increasing concentrations of Cdh23 EC1–2 in the cell‐culture media result in faster migration (Fig. 6). Faster migration in the presence of free Cdh23 EC1–2 (mimicking the soluble isoforms) proteins suggests that the cancer cells can facilitate migration by decreasing Cdh23 IS1 expression by promoter methylation and by the expression of soluble isoforms that interfere with homodimer formation.

Moreover, silencing of both Ecdh and Cdh23 resulted in increased cell migration and significant cell disaggregation, suggesting both can have a synergistic effect on cancer cell migration (Fig. 5). To verify that the increased cell migration and aggregation are not due to increases in cell proliferation or decreases in cell viability in siRNA‐transfected cells, we performed various proliferation assays, e.g. MTT assay, ATP cell viability assay, and cell‐cycle analysis; we observed that there was no significant change in cell proliferation or cell viability in these cells (Fig. S6). Cdh23 does not have a cytoplasmic β‐catenin binding domain. However, Cdh23 is known to interact directly with MAGI‐1, a member of the family of membrane‐associated guanylate kinases (Xu *et al*., 2008). MAGI‐1 can bind to β‐catenin via its PDZ5 domain, suggesting that MAGI‐1 may act as a bridge between Cdh23 and β‐catenin (Dobrosotskaya and James, 2000). Classical cadherins are known to modulate downstream signaling by β‐catenin and to regulate metastasis (Maretzky *et al*., 2005; Tucci *et al*., 2012). β‐Catenin is also a well‐known modulator of WNT pathway with a role in cancer cell proliferation and metastasis (Zhan *et al*., 2016). Silencing of Cdh23 in A549 cell lines showed no significant effect on the canonical β‐catenin pathway (Fig. S8).

## 5 Conclusion

Cdh23 is uniformly expressed in different tissues and mediates strong cell–cell adhesion junctions. To the best of our knowledge, the Cdh23‐mediated cell–cell junction is unique, where a non‐classical cadherin with more than five EC domains may be engaged in homophilic interactions. It is down‐regulated in cancer and produces soluble isoforms which disrupt the cell–cell attachment, leading to increased migration. Case studies with patients showed a strong correlation with patient survival, metastasis, and Cdh23 expression. Our data from experiments and data‐mining indicate that Cdh23 can potentially inhibit the spread of cancer to lymph nodes and metastasis. However, further studies are needed on immunocompromised/transgenic mice models injected with Cdh23 over‐expressed/down‐regulated cells to confirm the potential of Cdh23 as a metastasis suppressor. In addition, Cdh23 knockout mice (IMPC, MGI:1890219) featured multiple phenotypic deviations including partial pre‐weaning lethality, abnormal defecation, decreased bodyweight/mass, and abnormal blood chemistry (Dickinson *et al*., 2016).

## Conflict of interest

The authors declare no conflict of interest.

## Author contributions

SR supervised the project. MKS and SR designed the project and wrote the manuscript. MKS performed all the cell‐migration and cell‐aggregation assays. SS performed the imaging and maintained the cell lines. ND and MKS performed the bioinformatics.

## Supporting information


**Fig. S1.** Cadherin‐23 is well expressed in different normal tissues and down‐regulated in cancer.
**Fig. S2.** Immunohistochemical staining of tissues (*cf*. Fig. 1).
**Fig. S3.** Sorting and analysis of aggregation index of HEK293T cells transfected by Cdh23‐siRNA (*cf*. Fig. 2).
**Fig. S4.** Treatment of A549 cells with small‐molecule epigenetic modulators (*cf*. Fig. 4).
**Fig. S5.** Differential promoter methylation and patient survival analysis (*cf*. Fig. 4).
**Fig. S6.** Analysis of characteristics of different cancer cells suggest silencing Cdh23 expression has no effect on cell proliferation, cycle or membrane toxicity (*cf*. Fig. 5).
**Fig. S7.** Hanging‐drop analysis on cancer cell lines (*cf*. Fig. 5).
**Fig. S8.** Downstream targets of Cdh23 (*cf*. Fig. 5).
**Fig. S9.** Various isoforms of Cdh23 expressed in cells (*cf*. Fig. 6).
**Table S1.** Primers for mRNAs isoforms of Cdh23.
**Table S2.** qRT‐PCR plots for Cdh23 mRNA.
**Table S3.** Patient survival analysis as observed in Human Protein Atlas database.

**Data S1.** An excel file containing data downloaded from TCGA cbioportal (http://www.cbioportal.org/), TCGA Wanderer (http://maplab.imppc.org/wanderer/), and MethHC (http://methhc.mbc.nctu.edu.tw/php/index.php) used for analysis for LUAD, LUSC, and ESCC.
