# Supplementary material for: The strong propensity of Cadherin‐23 for aggregation inhibits cell migration
Source: Mol Oncol. 2019 Mar 19;13(5):1092–109. doi: 10.1002/1878-0261.12469 (PMC6487693; doi:10.1002/1878-0261.12469)
Supplement: Supplementary file 1 — Fig. S1. Cadherin‐23 is well expressed in different normal tissues and down‐regulated in cancer. Fig. S2. Immunohistochemical staining of tissues (cf. Fig. 1). Fig. S3. Sorting and analysis of aggregation index of HEK293T cells transfected by Cdh23‐siRNA (cf. Fig. 2). Fig. S4. Treatment of A549 cells with small‐molecule epigenetic modulators (cf. Fig. 4). Fig. S5. Differential promoter methylation and patient survival analysis (cf. Fig. 4). Fig. S6. Analysis of characteristics of different cancer cells suggest silencing Cdh23 expression has no effect on cell proliferation, cycle or membrane toxicity (cf. Fig. 5). Fig. S7. Hanging‐drop analysis on cancer cell lines (cf. Fig. 5). Fig. S8. Downstream targets of Cdh23 (cf. Fig. 5). Fig. S9. Various isoforms of Cdh23 expressed in cells (cf. Fig. 6). Table S1. Primers for mRNAs isoforms of Cdh23. Table S2. qRT‐PCR plots for Cdh23 mRNA. Table S3. Patient survival analysis as observed in Human Protein Atlas database. [file MOL2-13-1092-s001.pdf]

**The strong propensity of Cadherin-23 for aggregation inhibits cell migration**

**Malay K Sannigrahi[2], Cheerneni S. Srinivas[2], Nilesh Deokate[3], and Sabyasachi Rakshit[1,2]**

**1: Centre for Protein Science Design and Engineering, 2: Department of Chemical Sciences, 3: Department of Biological Sciences, Indian Institute of Science Education and Research Mohali, Punjab-140306**

**SUPPLEMENTARY INFORMATION:**

**INDEX**

Figure S1: Cdh23 is well expressed in different normal tissues and down-regulated in cancer

Figure S2: Immuno-histochemical Staining of tissues (In support of Figure 1)

Figure S3: Sorting and analysis of aggregation index of HEK293T cells transfected by Cdh23 siRNA (In support of Figure 2)

Figure S4: Treatment of A549 cells with small-molecule epigenetic modulators (In support of Figure 4)

Figure S5: Differential promoter methylation and patient survival analysis (In support of Figure 4)

Figure S6: Analysis of characteristics of different cancer cells suggest silencing Cdh23 expression has no effect on cell proliferation, cycle and membrane toxicity (In support of Figure 5)

Figure S7: Hanging Drop Analysis on cancer cell lines (In support of Figure 5)

Figure S8: Down-stream targets of Cdh23 (In support of Figure 5)

Figure S9: Various isoforms of Cdh23 expressed in cells (In support of Figure 6)

Supplementary Table 1: Primers for mRNAs isoforms of Cdh23

Supplementary Table 2: qRT-PCR plots for Cdh23

Supplementary Table 3: Patient survival analysis as observed in the Human Protein Atlas database

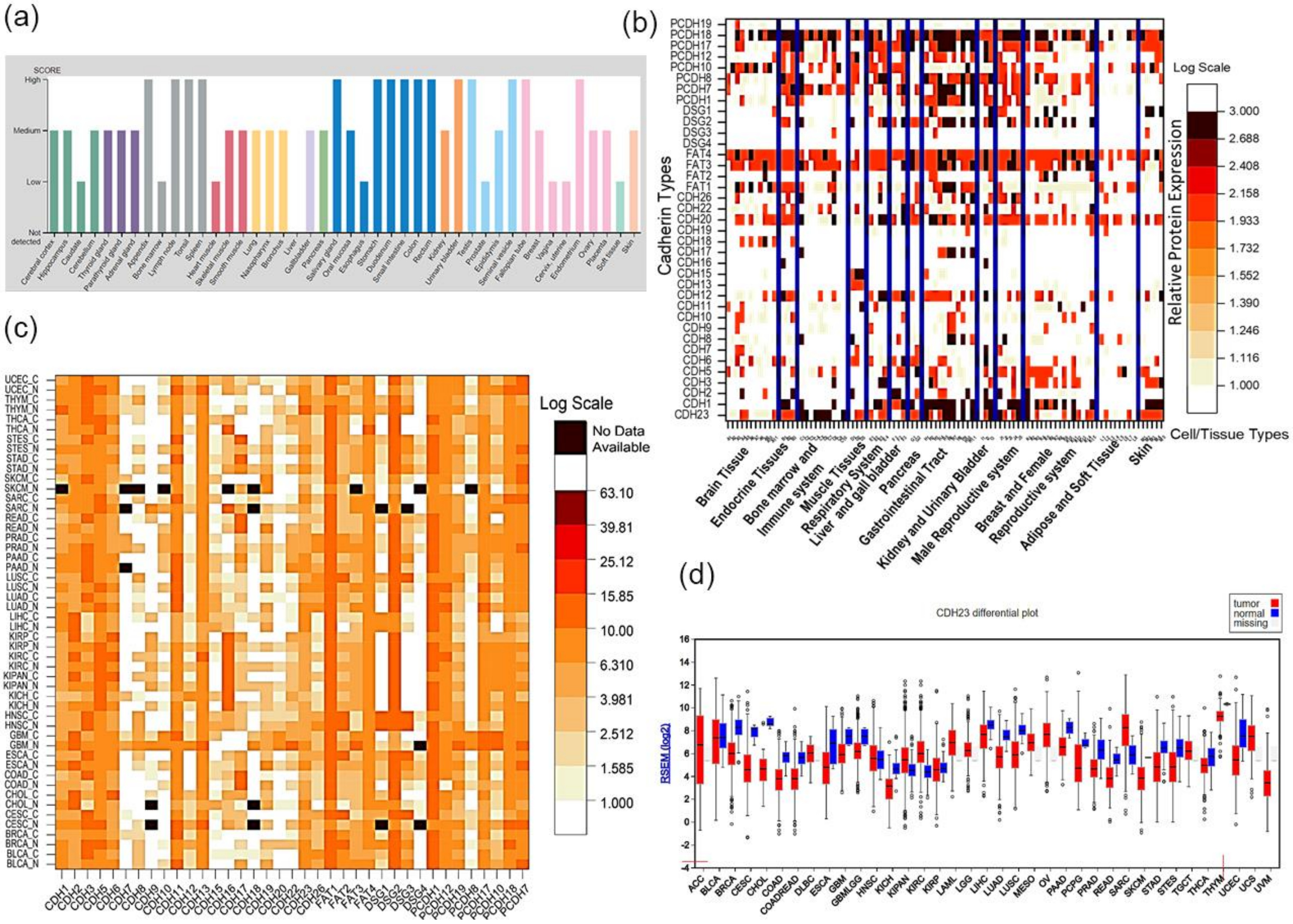


**Figure S1: Cdh23 is well expressed in different normal tissues and down-regulated in cancer** (a) The chart summarizes the differential expression of the Cdh23 protein in various tissues as reported in The Human Proteome Atlas (THPA)[1] database ( https://www.proteinatlas.org/search/CDH23). (b) The relative expression patterns of selected cadherins in various normal human tissues are represented in a heat-map (https://www.proteinatlas.org, staining intensity expressed as 2 for High, 1 for Low and 0 for no staining). (c) Heat-map showing mRNA expression (http://firebrowse.org/) patterns of some of the cadherin class of proteins ( in X-axis) in cancer and normal patients (in Y-axis); and (d) Cdh23 mRNA expression (http://firebrowse.org/viewGene.html?gene=cdh23) in different cancer types.

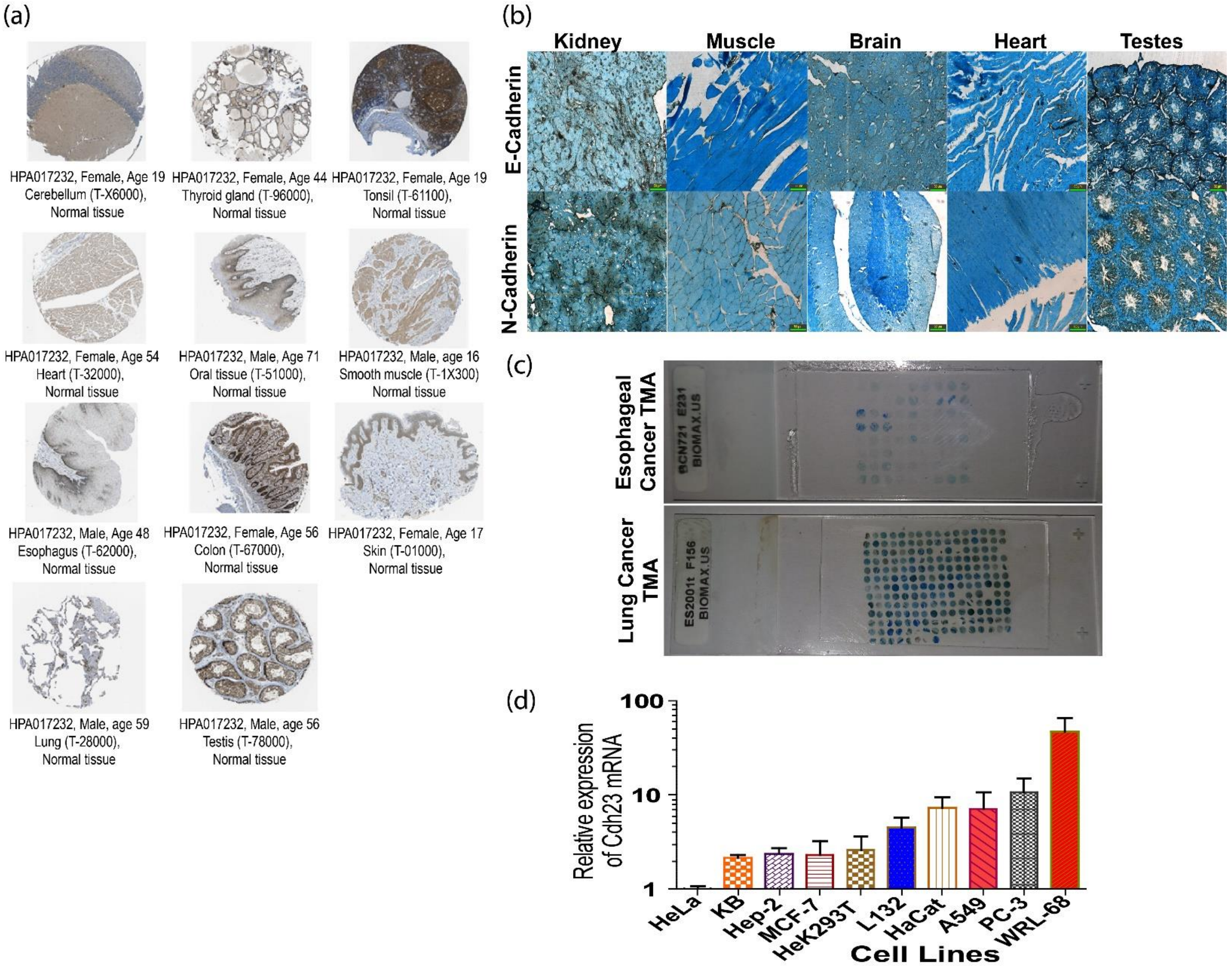


**Figure S2: Immunohistochemical staining of tissues *(In support to Figure 1)*** (a) Immunohistochemical (IHC) staining of different human tissues as deposited in THPA (https://www.proteinatlas.org/ENSG00000107736-CDH23/tissue). The staining was done using the same antibody as we used (Cdh23 antibody, HPA017232, Sigma). (b) Differential and distinct expression of E-cadherin(Ecdh) and N-cadherin(Ncdh) are shown in various mice tissues using IHC. In reference to Figure 1b (main text), we observed the nearly equal distribution of all three cadherins (Ecdh, Ncdh, and Cdh23) in the kidney. In muscle, distribution of Ncdh and Cdh23 are comparable with each other, and more than Ecdh. Expression of Ecdh is better in the brain than Ncdh and Cdh23, whereas, in Heart, none of the proteins were stained distinctly. Interestingly in testes, all three cadherins show better expression and the expression of Cdh23 is spatially distinct from Ecdh and Ncdh. (c) Along with mice tissues, we also performed staining on Human Tissue arrays. Two tissue microarray slides were stained: LC1005a-**Lung cancer** progression tissue array, including TNM, clinical stage and pathology grade; and ES2001-**Esophagus squamous cell carcinoma** and metastatic carcinoma tissue array, with adjacent tissue and adjacent normal tissue. Tissues were stained using Cdh23 antibody (HPA017232, Sigma) and Dako REAL EnVision Detection System kit (K500711, DAKO). The stained slides were analyzed using IHC Profiler (Figure 1c in main text) (d) Differential expression of Cdh23 mRNA in various cell lines (expressed as mean ± SEM) obtained from NCCS, Pune.

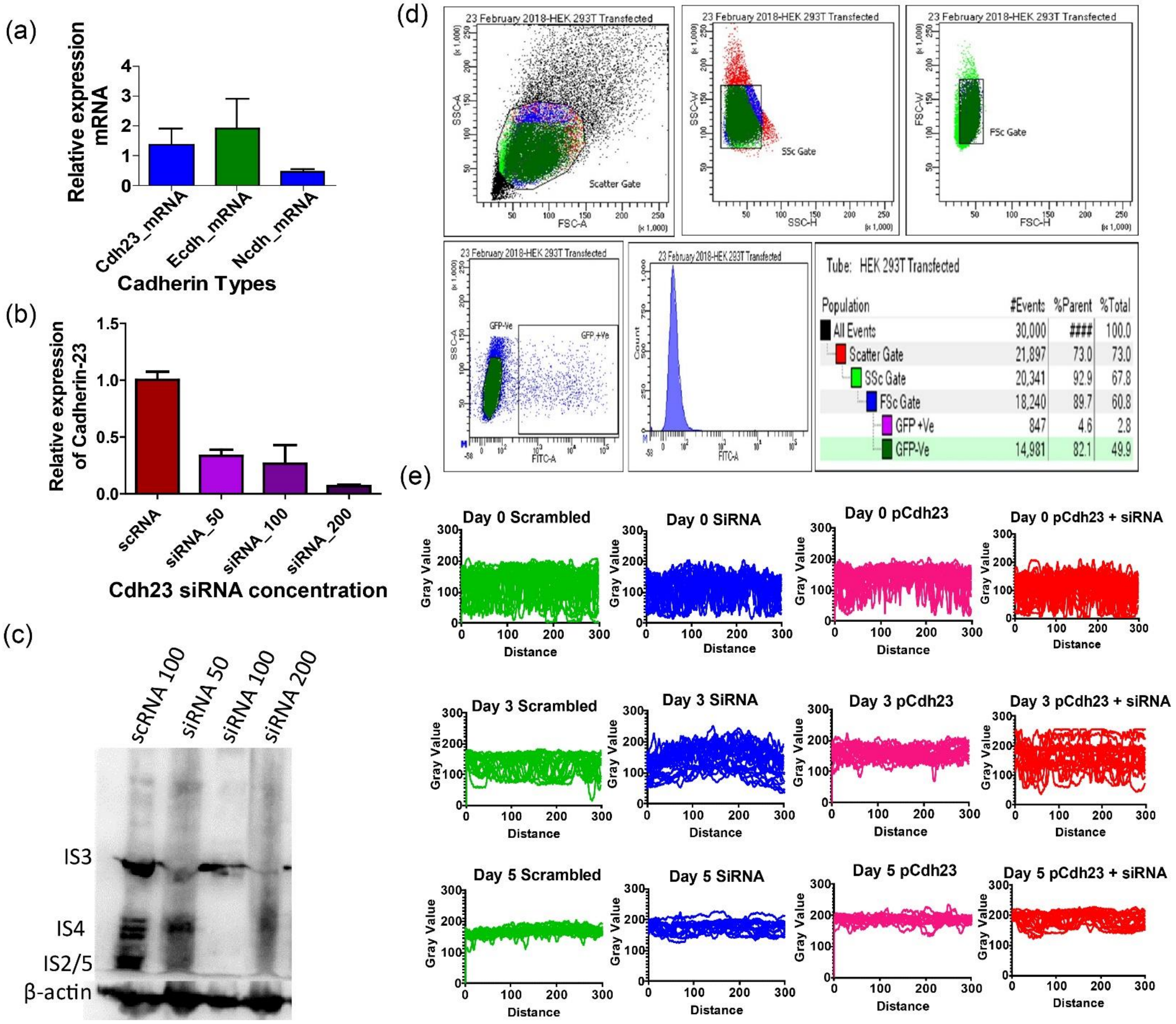


**Figure S3: Quantitative analysis of the aggregation of pCdh23-GFP transfected HEK293T cells *(In support of Figure 2)***: (a) qRT-PCR showed equivalent expression of Ecdh-mRNA, Ncdh-mRNA, and Cdh23-mRNA in HEK293T cells (expressed as mean±SEM). This experiment was performed to use HEK293T cells as model cell-line for our assays. (b) qRT-PCR showed dose-dependent down-regulation of Cdh23-mRNA expression on transfection of Cdh23 siRNA (c) Western blot reveals downregulation of Cdh23 proteins on siRNA transfection (d) pCdh23-GFP plasmid was transfected to 70% confluent culture of HEK293T cells using Lipofectamine 2000 and kept overnight to obtain expression of GFP. Then they were sorted with FACS to increase the number of transfected cells. Around $5X10^4$ were collected, grown overnight and used for different experiments; (e) Using ImageJ a straight line of 300-unit length was drawn a line-of-reference (lor) at the center of the cell clumps. Density and distribution of the cells were measured on the lor using Plot Profile function in Image J and plotted as "column average plot," where the X-axis represents the lor and the Y-axis the vertically averaged pixel intensity (Gray value). The AUC (Area Under the Curve) curves of days (period after transfection) as shown above were used to calculate mean (M) ± standard deviation (SD) for each. Finally, Relative standard deviation (RSD) was estimated from here (RSD = SD*100/M). (Mean±SEM were compared using two-tailed Mann Whitney Test)

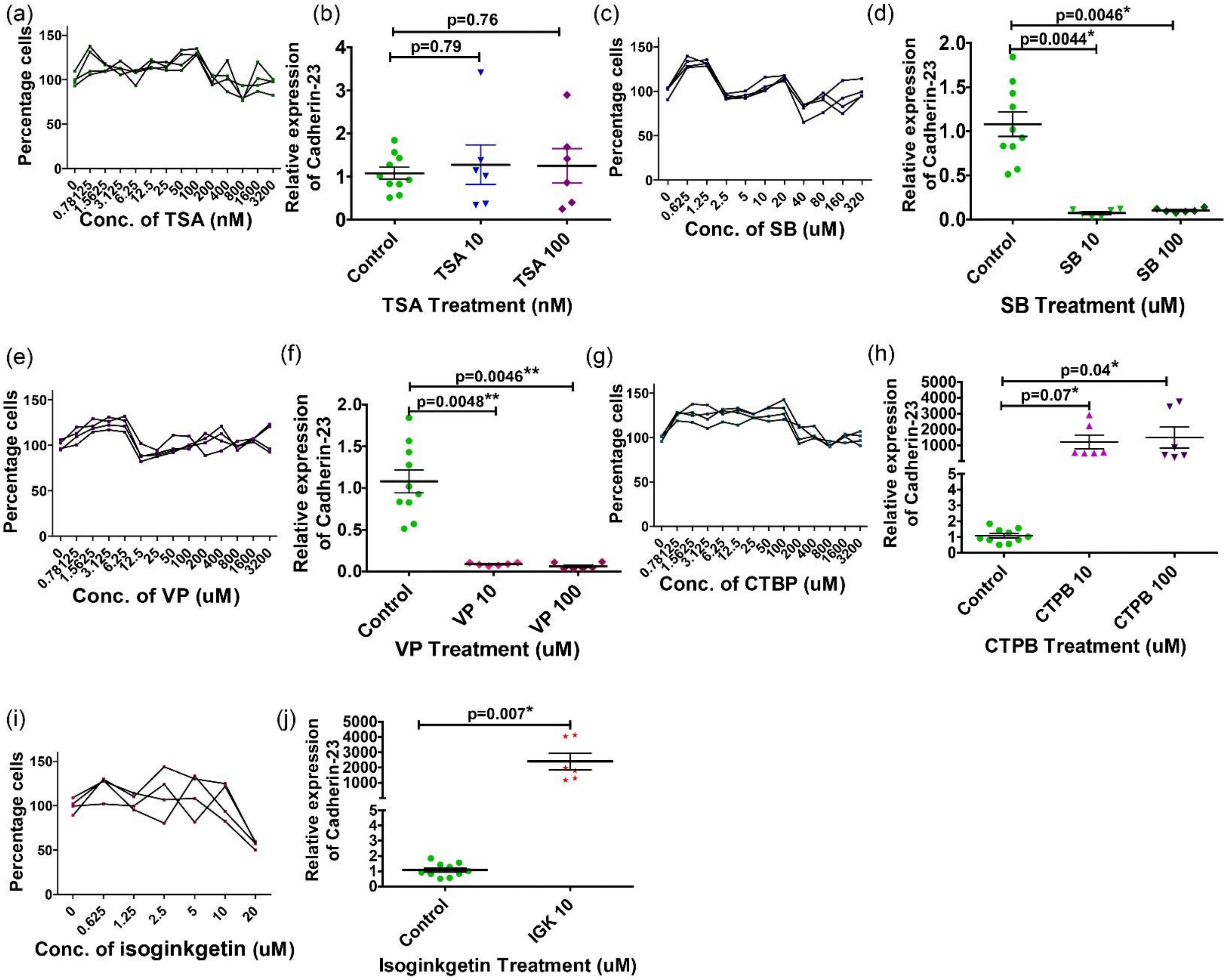


**Figure S4: Treatment of A549 cells with small-molecule epigenetic modulators *(In support of Figure 4)*:** Dose-response curves showing the viability of A549 cells with increasing concentration of (a) HDAC inhibitor (4 repeat experiments), Trichostatin A (TSA); (c) HDAC inhibitor, Sodium Butyrate (SB); (e) HDAC inhibitor, Valproic Acid (VP); (g) HAT Activator, N-[4-chloro-3-(trifluoromethyl)phenyl]-2-ethoxy-6-pentadecyl (CTPB); (i) Pre-mRNA Splicing Inhibitor, Isoginkgetin; w.r.t 100% viability at no drug (0). Figures b, d, f, h, j showed the relative expression of Cdh23 mRNA measured by qRT-PCR after the treatment of TSA (0, 10, 100 nM), SB (0, 10, 100 μM), VP (0,10, 100 μM), CTPB (0,10,100 μM), Isoginkgetin (0, 10 μM) respectively. Figure (b) showed no alteration in Cdh23 IS1 expression after TSA treatment; Figures (d) and (f) showed no restoration of Cdh23 IS1 expression after SB and VP treatment respectively. The revival of Cdh23 IS1 expression was observed after (h) CTBP, and, (j) Isoginkgetin treatment. CTBP is a potent activator of p300 HAT (histone acetyltransferase) which is known to modulate splicing. (In all cases, relative to mean control Cdh23IS1 expression, Mean ±SEM values were compared w.r.t control using two-tailed paired t test)

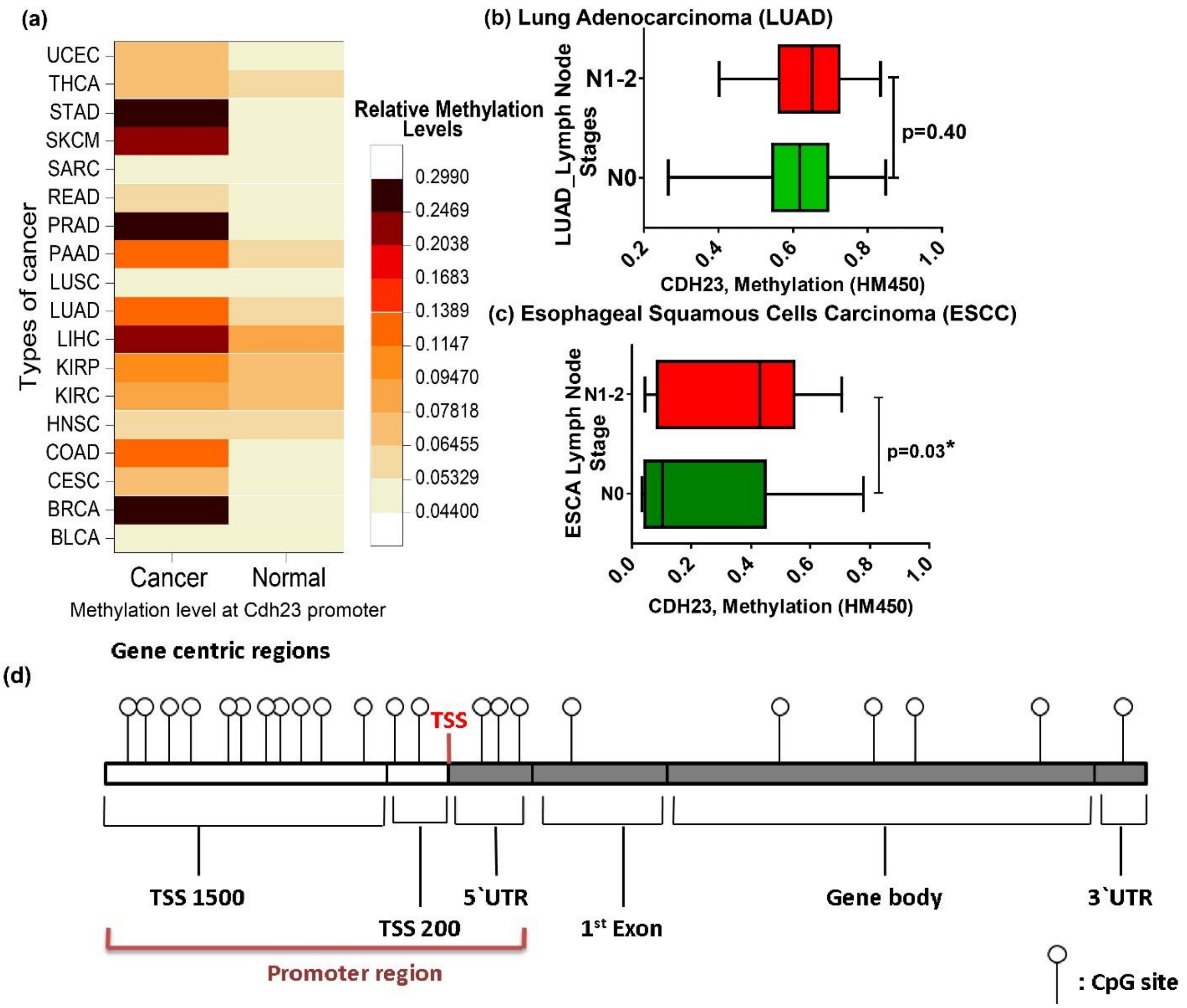


**Figure S5: Heat-map analysis of the promoter-methylation for Cdh23 in various cancers and its correlation with cancer metastasis *(In support of Figure 4).*** (a) A heat-map comparison of mean methylation levels of Cdh23 promoter (NM_022124_promoter_mean) in different cancers (http://methhc.mbc.nctu.edu.tw/php/index.php) shows increased level of promoter methylation in cancer compared to respective normal controls; Increased methylation was observed in higher nodal stage (N1-2) compared to N0 stage in (b) Lung Adenocarcinoma, (c) Esophageal Squamous cell carcinoma (Mean ±SD values were compared using two-tailed Mann Whitney Test) as observed in cbioportal ( http://www.cbioportal.org/) and (d) Diagrammatic representation of various regions as observed in MethHC.

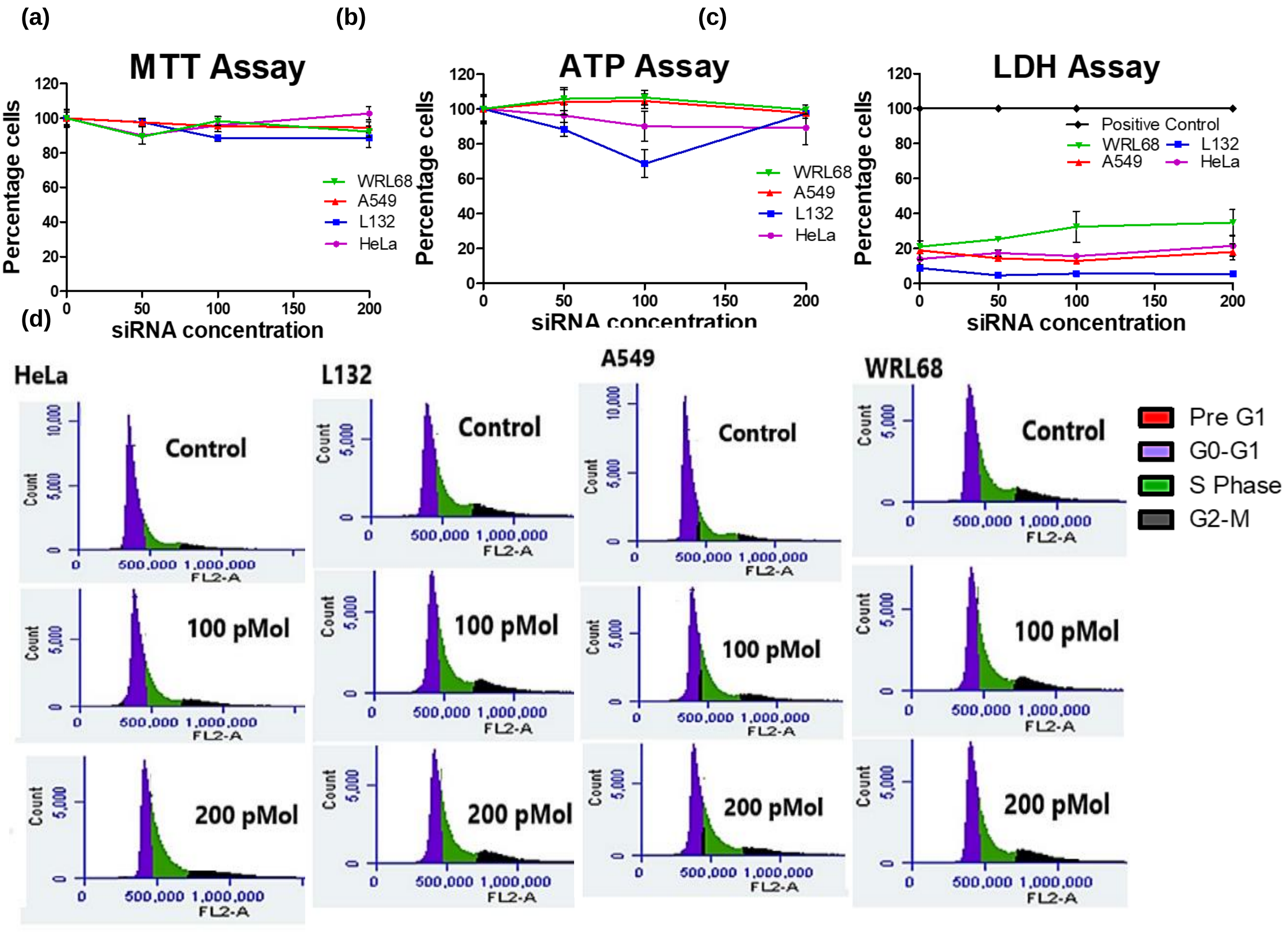


**Figure S6**: **Studies with different cancer cells suggest silencing Cdh23 expression has no effect on cell proliferation, cycle and membrane toxicity** ***(In support of Figure 5)***: (a) No significant change was observed in cell proliferation by MTT assay in different cancer cell lines when scrambled control (Hela, 100±8.10; L132, 100±6.13; A549, 100±9.12; WRL68, 100±2.81) was compared to increasing Cdh23 siRNA concentration from 50 pMol (Hela, 90.03±8.71; L132, 97.74±3.16; A549, 97.68±3.79; WRL68, 89.4±1.7) to 100 pMol (Hela, 95.86±3.26; L132, 88.58±3.25; A549, 95.32±5.48; WRL68, 98.43±4.27) to 200 pMol (Hela, 102.74±6.65; L132, 88.61±9.81; A549, 94.69±5.36; WRL68, 92.26±5.30) respectively; (b) No consistent change was observed on ATP cell viability assay of these cell lines (50 pMol, 98.56±6.96; 100 pMol, 92.44±15.15; 200 pMol, 95.98±4.04) when compared to scrambled control (100); (c) No significant release of LDH was observed on an average in these cell lines with increasing concentration of siRNA (50 pMol, 15.45±7.42; 100 pMol, 16.65±9.8; 200 pMol, 19.99±10.47) compared to scrambled control (15.73±10.47); and, lysed cells as positive control (100); (d) No significant changes were also observed in distribution of cells (expressed as percentage of cells on an average of the four cell lines) at different cell cycle phases at 100 pMol (Pre G1, 0.37±0.13; G0-G1, 51.4±4.8; S-Phase, 21.55±3.38; G2-M, 16.36±1.6) and 200 pMol (Pre G1, 0.32±0.05; G0-G1, 47.8±2.6; S-Phase, 31.33±3.39; G2-M, 18.44±1.54) compared to scrambled control (Pre G1, 0.45±0.07; G0-G1, 61±8.6; S-Phase, 22.86±4.68; G2-M, 13.4±3.9). (for each case Mean ± SD values for each time point was compared w.r.t scrambled control using two-tailed paired t test)

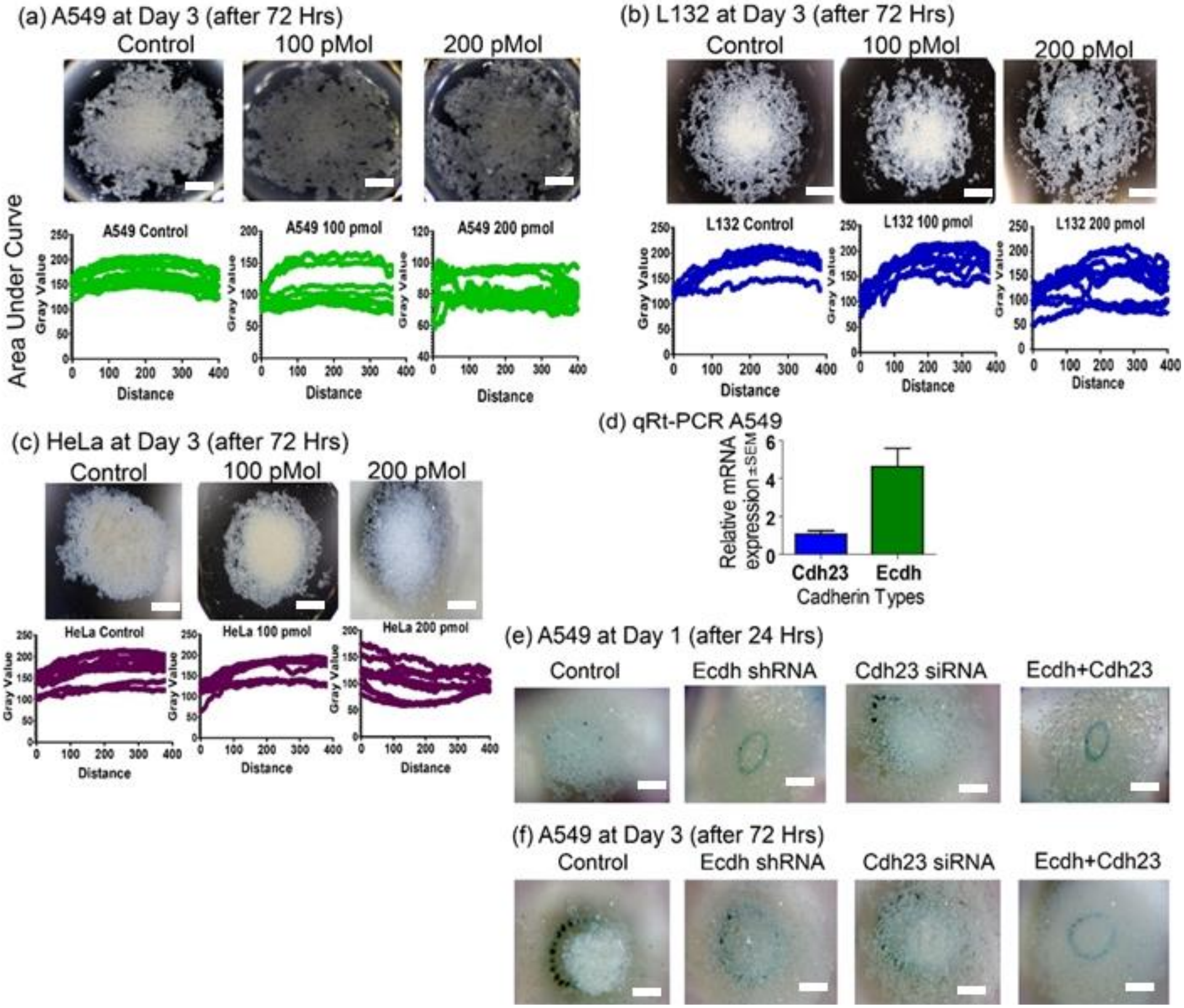


**Figure S7**: **Modified Hanging-drop assay on different cancer cell lines *(In support of Figure 5)*:** Plots of Area under the curve after transfected with scrambled control (200 pMol) and Cdh23 siRNA (100 pMol and 200 pMol) for 3 days (72 hrs) for Hanging-drop assay in cancer cell lines (a) A549, (b) L132 and (c) HeLa, (d) Relative mRNA expression of Cdh23 and Ecdh in A549 cells (relative to Cdh23 expression, expressed as Mean±SEM), Modified hanging drop assay was performed on A549 cells transfected with 200 pMol Scrambled control (Control), 200 pMol of Cadherin-23 (Cdh23 siRNA), 2 ng of E-cadherin shRNA (Ecdh shRNA), and, both (Cdh23+eCdh) and representative pictures (e) at Day 1 (24 hrs) and, (f) at Day 3 (72 hrs). (4X images, Scale bar=1.2mm)

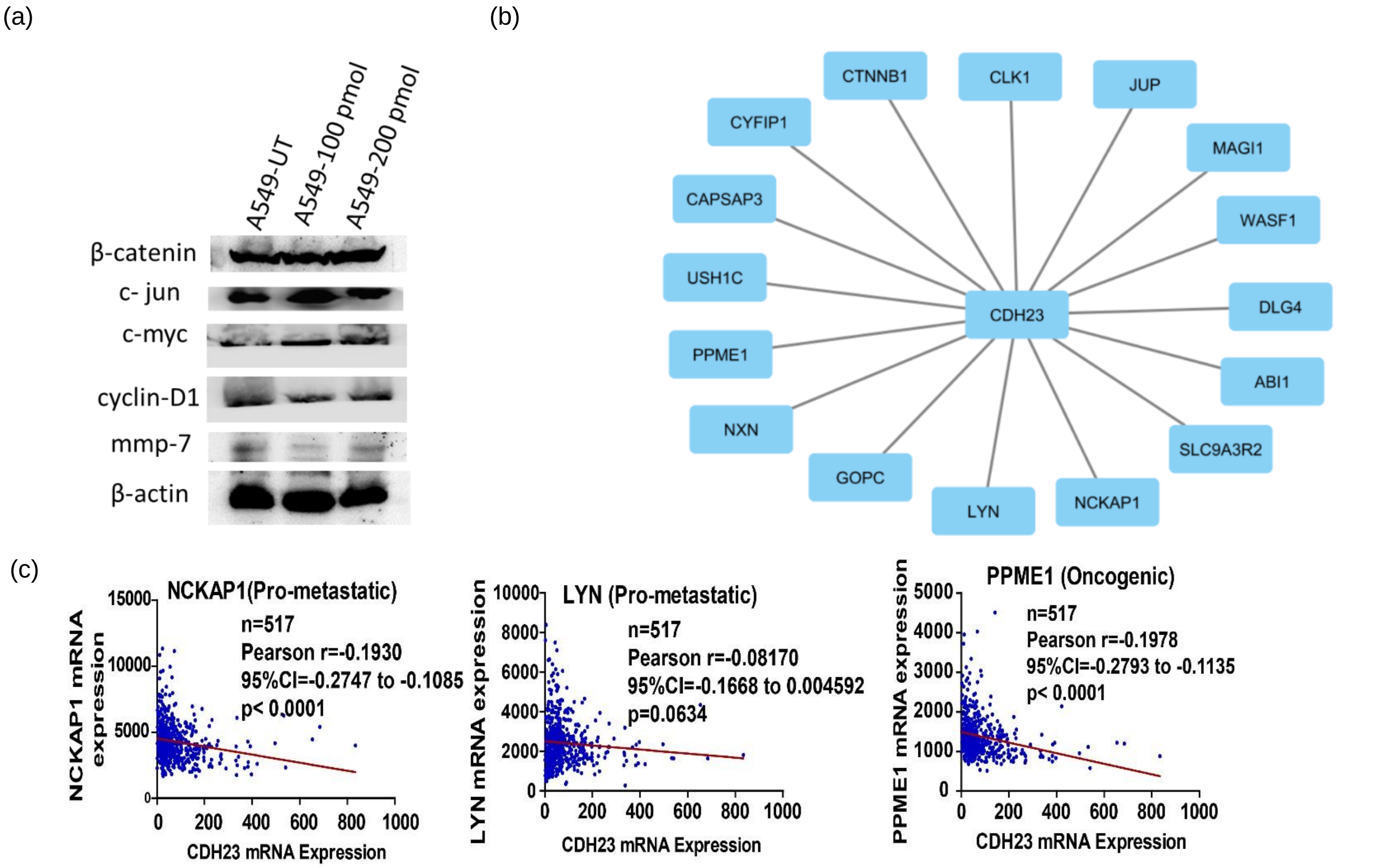


**Figure S8: Down-stream targets of Cdh23 *(In support of Figure 5)*:** (a) Silencing Cdh23 using Cdh23-siRNA (for 48 hrs) does not show any significant change in canonical β-catenin pathway; (b) Based on Biogrid 3.4,[2] Mentha[3] and String[4] analysis, Cdh23 interacts with many other proteins; (c) TCGA analysis of correlation of their expression shows negative correlation with pro-metastatic (NCKAP1[5] and LYN[6]) and oncogenic (PPME1[7]).

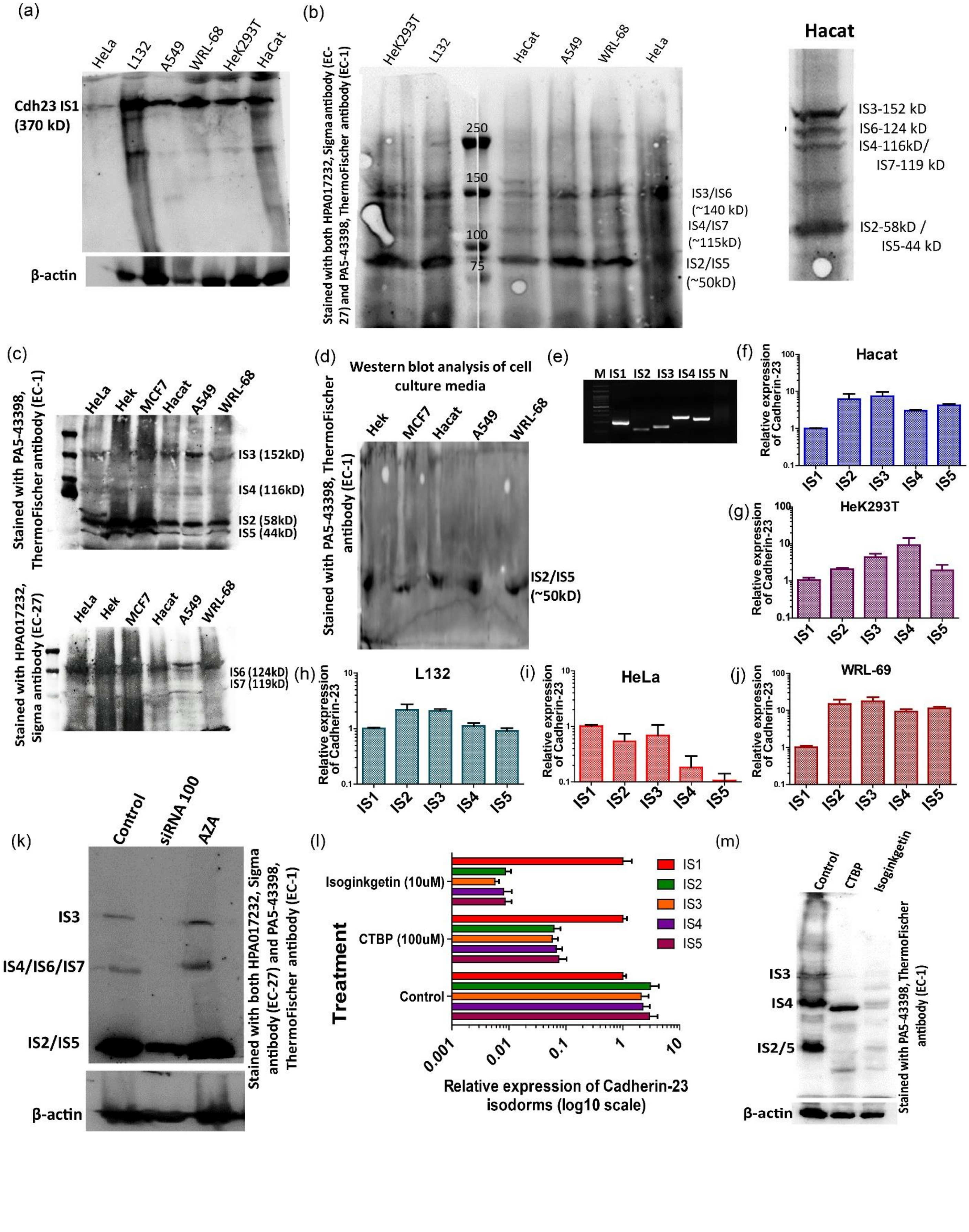
(a)
HeLa L132 A549 WRL-68 HeK293T HaCat
Cdh23 IS1 (370 kD)
β-actin
(b)
HeK293T L132 HaCat A549 WRL-68 HeLa
Stained with both HPA017232, Sigma antibody (EC-27) and PA5-43398, ThermoFischer antibody (EC-1)
250
150
100
75
IS3/IS6 (~140 kD)
IS4/IS7 (~115kD)
IS2/IS5 (~50kD)
Hacat
IS3-152 kD
IS6-124 kD
IS4-116kD/ IS7-119 kD
IS2-58kD / IS5-44 kD
(c)
Stained with PA5-43398, ThermoFischer antibody (EC-1)
HeLa Hek MCF7 Hacat A549 WRL-68
IS3 (152kD)
IS4 (116kD)
IS2 (58kD)
IS5 (44kD)
Stained with HPA017232, Sigma antibody (EC-27)
IS6 (124kD)
IS7 (119kD)
(d)
Western blot analysis of cell culture media
Stained with PA5-43398, ThermoFischer antibody (EC-1)
Hek MCF7 Hacat A549 WRL-68
IS2/IS5 (~50kD)
(e)
M IS1 IS2 IS3 IS4 IS5 N
(f) Hacat
(g) HeK293T
(h) L132
(i) HeLa
(j) WRL-69
Relative expression of Cadherin-23
IS1 IS2 IS3 IS4 IS5
(k)
Control siRNA 100 AZA
IS3
IS4/IS6/IS7
IS2/IS5
β-actin
Stained with both HPA017232, Sigma antibody (EC-27) and PA5-43398, ThermoFischer antibody (EC-1)
(l)
Treatment
Isoginkgetin (10uM)
CTBP (100uM)
Control
IS1 IS2 IS3 IS4 IS5
Relative expression of Cadherin-23 isodorms (log10 scale)
(m)
Control CTBP Isoginkgetin
IS3
IS4
IS2/5
β-actin
Stained with PA5-43398, ThermoFischer antibody (EC-1)

**Figure S9: Various isoforms of Cdh23 expressed in cancer cells *(In support of Figure 6)***: Western Blot of different cell lines for (a) Cdh23 isoform 1 (370 kD) in 5% SDS gel, (b) Other cadherin isoforms (Cdh23 IS2 to IS7) in 10% SDS gel, (c) Western blot of media [10 ml collected and concentrated to 1 ml] using Cdh23-EC1 targeted antibody (for IS2, 3, 4 and 5 with no transmembrane domain) in 10% SDS gel, (d) using Cdh23-EC1 targeted antiboby (for IS2, 3, 4 and 5) and Cdh23-EC27 targeted antibody (for IS3, 6 and 7) in 10% SDS gel; shows expression of various Cdh23 isoforms at protein level, (e) Representative figure of Cdh23 isoforms (Isoform 1 to 5) mRNA qRT-PCR bands in 1.5% agarose with relative level of expression in (f) Hacat, (g) HeK293T, (h) L132, (i) HeLa, (j) WRL-68 shows expression of different isoforms at mRNA level; (k) Western blot of A549 cells were transfected with Cdh23 siRNA (100 pMol) and AZA (50 nM) for 48 hrs shows targeting of Cdh23 isoforms by Cdh23 siRNA, (l) Relative mRNA expression of Cdh23 isoforms by qRT-PCR (relative to mean Isoform-1 expression, expressed as Mean±SEM) and (m) Protein expression by western blot in 10% SDS gel after treatment with CTBP, activator of p300 HAT (histone acetyltransferase, 100 µM) and RNA splicing inhibitor, isoginkgetin (10 µM) for 48 hrs shows decreased mRNA expression (relative to mean Isoform-1 expression, expressed in log10 scale as Mean±SEM) and gradual protein degradation of Cdh23 isoforms on targeting the cell splicosome machinery.

**Supplementary Table 1: Primers for mRNAs isoforms of Cdh23**

| mRNA isoform | | Sequence | Product length | Tm |
|---|---|---|---|---|
| NM_022124.5 Homo sapiens cadherin related 23 (CDH23), transcript variant 1, mRNA (CDH IS1) | Forward primer | TCAACACCAGCCTCATCACC | 151 | 60 |
| | Reverse primer | TAGTCCAGCTCTTTGGCAGC | | |
| NM_052836.3 Homo sapiens cadherin related 23 (CDH23), transcript variant 2, mRNA (CDH IS2) | Forward primer | AGAAGGGCAACCCTCTCCAT | 108 | 60 |
| | Reverse primer | CCTTGTCAAAGGGCAAGTCC | | |
| NM_001171930.1 Homo sapiens cadherin related 23 (CDH23), transcript variant 3, mRNA (CDH IS3) | Forward primer | GTACCGCTTCAACGCCTACA | 133 | 60 |
| | Reverse primer | ACACAGAAGGAGCTCAACCA | | |
| NM_001171931.1 Homo sapiens cadherin related 23 (CDH23), transcript variant 4, mRNA (CDH IS4) | Forward primer | TGTTGGGCTTTAGCCTCTGG | 250 | 60 |
| | Reverse primer | GGCCACATTCCTCCAAGTCA | | |
| NM_001171932.1 Homo sapiens cadherin related 23 (CDH23), transcript variant 5, mRNA (CDH IS5) | Forward primer | CAATGACAATGCCCCGGAGT | 223 | 60 |
| | Reverse primer | GGTGCCGCAATGTTTGTTTCT | | |
| 18S Internal Control | Forward primer | CGCGGTTCTATTTTGTTGGT | 219 | 60 |
| | Reverse primer | AGTCGGCATCGTTTATGGTC | | |

**Supplementary Table 2: qRT-PCR plots for Cdh23**

| | Amplification Plot | Melt Curve | Melt Peak |
|---|---|---|---|
| **NM_022124.5 Homo sapiens cadherin related 23 (CDH23), transcript variant 1, mRNA** | 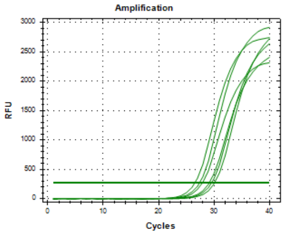 | 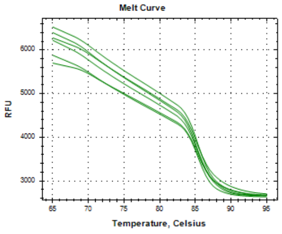 | 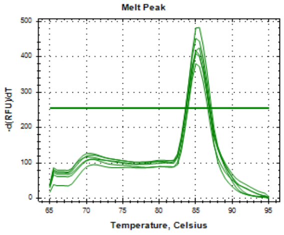 |
| **NM_052836.3 Homo sapiens cadherin related 23 (CDH23), transcript variant 2, mRNA** | 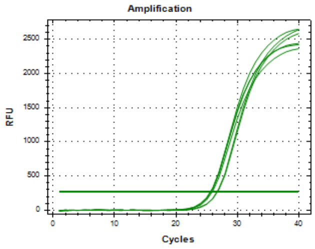 | 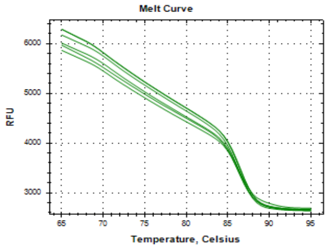 | 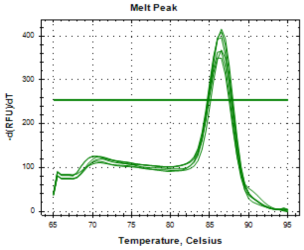 |
| **NM_001171930.1 Homo sapiens cadherin related 23 (CDH23), transcript variant 3, mRNA** | 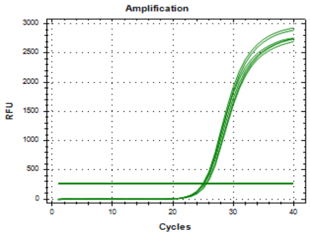 | 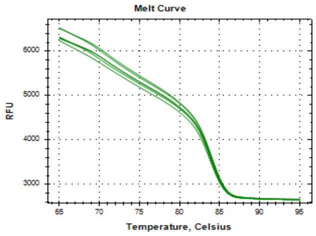 | 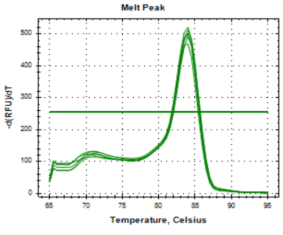 |
| **NM_001171931.1 Homo sapiens cadherin related 23 (CDH23), transcript variant 4, mRNA** | 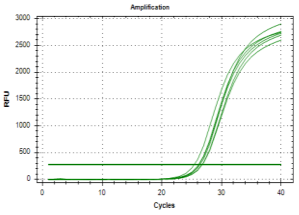 | 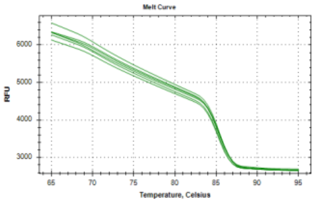 | 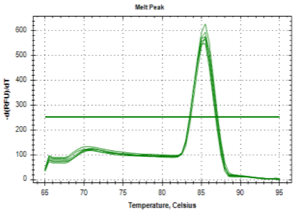 |
| **NM_001171932.1 Homo sapiens cadherin related 23 (CDH23), transcript variant 5, mRNA** | 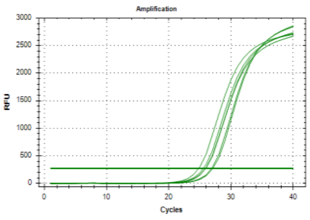 | 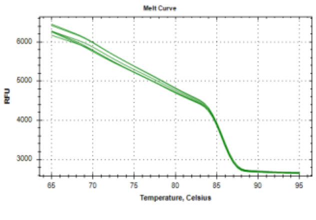 | 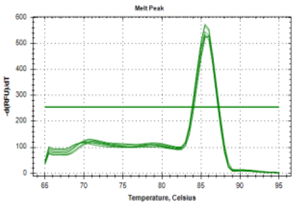 |
| **18S Internal Control** | 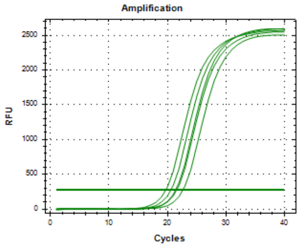 | 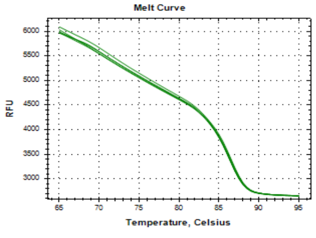 | 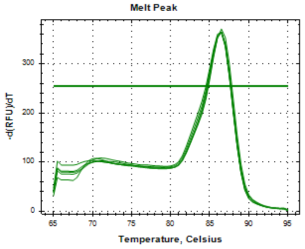 |

**Supplementary Table 3: Patient survival analysis** as observed in Human Protein Atlas database (https://www.proteinatlas.org/ENSG00000107736-CDH23/pathology) with mean expression cutoff analysis using fragments per kilobase of exon model per million mapped (FPKM) and percentage of patients survived having high and low expression than the cutoff. (CESC: Cervical Squamous Cell Carcinoma and Endocervical Adenocarcinoma, HNSC: Head and Neck Squamous Cell Carcinoma, LUAD: Lung Adenocarcinoma, UCEC: Uterine Corpus Endometrial Carcinoma, BRCA: Breast Invasive Carcinoma, SKCM: Skin Cutaneous Melanoma and LC: Liver Cancer)

| **Type of Cancer** | **CESC** | **HNSC** | **LUAD** | **UCEC** | **BRCA** | **SKCM$** | **LC$$** |
|---|---|---|---|---|---|---|---|
| **Expression cutoff** | 0.1 FPKM | 0.2 FPKM | 0.1 FPKM | 0.3 FPKM | 0.3 FPKM | 0.0 FPKM | 0.3 FPKM |
| **5-year survival on high Cdh23 expression** | 78% | 50% | 43% | 46% | 85% | 33% | 49% |
| **5-year survival on low Cdh23 expression** | 58% | 42% | 30% | 34% | 78% | 0% | 38% |
| **Log-rank P value** | **0.00286**** | 0.0552 | **0.0109**** | **0.00034***** | 0.0676 | **0.00644**** | 0.0793 |

$SKCM (Melanoma) values are for 3-year survival $$Liver Cancer

**Supplementary References:**


1. Uhlen, M.; Fagerberg, L.; Hallstrom, B. M.; Lindskog, C.; Oksvold, P.; Mardinoglu, A.; Sivertsson, A.; Kampf, C.; Sjostedt, E.; Asplund, A.; Olsson, I.; Edlund, K.; Lundberg, E.; Navani, S.; Szigyarto, C. A.; Odeberg, J.; Djureinovic, D.; Takanen, J. O.; Hober, S.; Alm, T.; Edqvist, P. H.; Berling, H.; Tegel, H.; Mulder, J.; Rockberg, J.; Nilsson, P.; Schwenk, J. M.; Hamsten, M.; von Feilitzen, K.; Forsberg, M.; Persson, L.; Johansson, F.; Zwahlen, M.; von Heijne, G.; Nielsen, J.; Ponten, F., Proteomics. Tissue-based map of the human proteome. *Science* **2015,** *347* (6220), 1260419.
2. Chatr-Aryamontri, A.; Oughtred, R.; Boucher, L.; Rust, J.; Chang, C.; Kolas, N. K.; O'Donnell, L.; Oster, S.; Theesfeld, C.; Sellam, A.; Stark, C.; Breitkreutz, B. J.; Dolinski, K.; Tyers, M., The BioGRID interaction database: 2017 update. *Nucleic acids research* **2017,** *45* (D1), D369-D379.
3. Calderone, A.; Castagnoli, L.; Cesareni, G., mentha: a resource for browsing integrated protein-interaction networks. *Nature methods* **2013,** *10* (8), 690-1.
4. von Mering, C.; Huynen, M.; Jaeggi, D.; Schmidt, S.; Bork, P.; Snel, B., STRING: a database of predicted functional associations between proteins. *Nucleic acids research* **2003,** *31* (1), 258-61.
5. Teng, Y.; Qin, H.; Bahassan, A.; Bendzunas, N. G.; Kennedy, E. J.; Cowell, J. K., The WASF3-NCKAP1-CYFIP1 Complex Is Essential for Breast Cancer Metastasis. *Cancer research* **2016,** *76* (17), 5133-42.
6. Guan, H.; Zhou, Z.; Gallick, G. E.; Jia, S. F.; Morales, J.; Sood, A. K.; Corey, S. J.; Kleinerman, E. S., Targeting Lyn inhibits tumor growth and metastasis in Ewing's sarcoma. *Molecular cancer therapeutics* **2008,** *7* (7), 1807-16.
7. Li, J.; Han, S.; Qian, Z.; Su, X.; Fan, S.; Fu, J.; Liu, Y.; Yin, X.; Gao, Z.; Zhang, J.; Yu, D. H.; Ji, Q., Genetic amplification of PPME1 in gastric and lung cancer and its potential as a novel therapeutic target. *Cancer biology & therapy* **2014,** *15* (1), 128-34.
